# Zinc in innate and adaptive tumor immunity

**DOI:** 10.1186/1479-5876-8-118

**Published:** 2010-11-18

**Authors:** Erica John, Thomas C Laskow, William J Buchser, Bruce R Pitt, Per H Basse, Lisa H Butterfield, Pawel Kalinski, Michael T Lotze

**Affiliations:** 1 Department of Surgery, University of Pittsburgh, 200 Lothrop Street, Pittsburgh, PA 15213, USA; 2 Department of Occupational Health, University of Pittsburgh, 100 Technology Drive, Pittsburgh, PA 15219, USA; 3 Department of Immunology, University of Pittsburgh, 200 Lothrop Street, Pittsburgh, PA 15213, USA; 4 Department of Medicine, University of Pittsburgh, 3550 Terrace Street, Pittsburgh, PA 15261, USA

## Abstract

Zinc is important. It is the second most abundant trace metal with 2-4 grams in humans. It is an essential trace element, critical for cell growth, development and differentiation, DNA synthesis, RNA transcription, cell division, and cell activation. Zinc deficiency has adverse consequences during embryogenesis and early childhood development, particularly on immune functioning. It is essential in members of all enzyme classes, including over 300 signaling molecules and transcription factors. Free zinc in immune and tumor cells is regulated by 14 distinct zinc importers (ZIP) and transporters (ZNT1-8). Zinc depletion induces cell death via apoptosis (or necrosis if apoptotic pathways are blocked) while sufficient zinc levels allows maintenance of autophagy. Cancer cells have upregulated zinc importers, and frequently increased zinc levels, which allow them to survive. Based on this novel synthesis, approaches which locally regulate zinc levels to promote survival of immune cells and/or induce tumor apoptosis are in order.

## 

"*Finding a potent role for zinc in the regulation of autophagic PCD establishes zinc deprivation as a universal cell death signal, regardless of which route of degradation--apoptotic or autophagic --is chosen by cells*." Andreas Helmersson, Sara von Arnold, and Peter V. Bozhkov. The Level of Free Intracellular Zinc Mediates Programmed Cell Death/Cell Survival Decisions in Plant Embryos. Plant Physiol. 2008 July; 147 (3): 1158-1167.

"*It's a business. If I could make more money down in the zinc mines I'd be mining zinc*." Roger Maris (American professional Baseball Player. 1934-1985)

"*We have everything but the kits in zinc*." Albert Donnenberg, PhD (Flow Cytometrist, UPSHS) 2009

## Biological Role of Zinc

Zinc is the second most abundant metal in organisms (second only to iron), with 2-4 grams distributed throughout the human body. Most zinc is found in the brain, muscle, bones, kidney, and liver, with the highest concentrations in the prostate and parts of the eye. It is the only metal that is a coenzyme to all enzyme classes [1-3]. A biologically critical role for zinc was first reported in 1869, when it was shown to be required for the growth of the fungus, Aspergillus niger [4]. In 1926, zinc was found to be required for the growth of plants [5], and shortly thereafter, its first function in animals was demonstrated [6-8]. Now, zinc has been shown to be important also in prokaryotes [9]. In the last half-century the consequences of zinc deficiency have been recognized.

Zinc is a biologically essential trace element; critical for cell growth, development and differentiation [10]. It is required for DNA synthesis, RNA transcription, cell division, and cell activation [11], and is an essential structural component of many proteins, including signaling enzymes and transcription factors. Zinc is required for the activity of more than 300 enzymes, interacting with zinc-binding domains such as zinc fingers, RING fingers, and LIM domains [12-14]. The RING finger domain is a zinc finger which contains a Cys3HisCys4 amino acid motif, binding two zincs, contains from 40 to 60 amino acids. RING is an acronym specifying Really Interesting New Gene. LIM domains are structural domains, composed of two zinc finger domains, separated by a two-amino acid residue hydrophobic linker. They were named following their discovery in the proteins Lin11, Isl-1 and Mec-3. LIM-domain proteins play roles in cytoskeletal organization, organ development and oncogenesis. More than 2000 transcription factors have structural requirements for zinc to bind DNA, thereby revealing a critical role for zinc in gene expression.

Zinc is required for both normal cell survival (as above) and for cell death via its role in apoptosis. We propose that zinc may also regulate autophagy and other forms of survival due to its early sensitivity to cell stress. Thus, zinc could play a central role, regulating apoptosis and autophagy as well as immune cell function. Cancer cells are continuously stressed (genomic stress, ER stress, nutrient stress, oxidant stress, etc) and selected for survival (likely by autophagy). Here we review the current studies surrounding zinc, and propose that zinc has a spectrum of effects on cell death and survival, where zinc depletion induces cell death via apoptosis (or necrosis if apoptotic pathways are blocked) while sufficient zinc levels allows maintenance of cell survival pathways such as autophagy and regulation of reactive oxygen species. Cancer cells have upregulated zinc importers, and most frequently increased zinc levels, which allow them to survive. Based on these notions, means to locally regulate zinc levels to promote survival of immune cells and promote tumor apoptosis are in order.

## Dietary Zinc and Deficiency

Red meat is the primary sources of zinc for most Americans. The already low amount of zinc in vegetables is further chelated by phytates and is therefore not as available for absorption. Nuts, and fruits, whole grain bread, dairy products, and fortified breakfast cereals are other sources of zinc. Oysters have the highest zinc per serving of any common food [15,16].

Zinc is taken up primarily in the proximal small intestine, and depends heavily on ZIP4. Once transported through the enterocytes and into the blood, zinc binds to albumin, transferrin, α-2 macroglobulin, and immunoglobulin G, and travels to the liver where the zinc is stored in hepatocytes until it is released back into the blood to again bind carrier molecules and travel to the tissues where zinc intake will be regulated by zinc import and transport proteins [17].

Over one billion people in developing countries are nutritionally deficient in zinc [18]. Zinc deficiency is associated with a range of pathological states, including skin changes, loss of hair, slowed growth, delayed wound healing, hypogonadism, impaired immunity, and brain development disorders [6,10,19], all of which are reversible with zinc supplementation. Zinc deficiencies occur as a result of malabsorption syndromes and other gastrointestinal disorders, chronic liver and renal diseases, sickle cell disease, excessive alcohol intake, malignancy, cystic fibrosis, pancreatic insufficiency, rheumatoid arthritis, and other chronic conditions [18,20-25]. In humans, acrodermatitis enteropathica-like eruptions are commonly found with zinc deficiency [26]. These pathological states and the associated zinc deficiencies are linked to increased infection and prolonged healing time, both of which are indicators of compromised immunity. In developing countries, previously pervasive conditions such as diarrhea [27] and lower respiratory illness [28] are associated with low zinc. Unfortunately, quantifying human zinc to identify deficiency and preventing zinc toxicity (due to excess supplementation) is an ongoing challenge [29]. These findings suggest a role for zinc in immune cell homeostasis *in vivo *[30,31].

## A Signaling Ion

Zinc may act as a signaling molecule, both extracellularly (as in neurotransmitters) and intracellularly (as in calcium second-messenger systems). In nerve cells, zinc can be found in membrane-enclosed synaptic vesicles, from which it is released via exocytosis to bind ligand gated ion channels (such as NMDA receptors, Ca2+-permeable AMPA/kainite receptors, and voltage-dependent Ca2+ channels (VDCC)), activating postsynaptic cells [32]. Additionally, changes in the concentration of intracellular free zinc control immune cell signal transduction by regulating the activity of major signaling molecules, including kinases (PKC, LCK), phosphatases (cyclic nucleotide phosphodiesterases and MAPK phosphatases), and transcription factors (NFkB).

In T cells, zinc treatment stimulates the kinase activity of PKC, its affinity to phorbol esters, and its binding to the plasma membrane and cytoskeleton [33], while zinc chelators inhibit the induction of these events [34]. Zinc ions also promote activation of LCK, a Src-family tyrosine kinase, and its recruitment to the T cell receptor complex [35]. The interaction of LCK with CD44 is also zinc dependent [36]. The release of zinc from lysosomes also appears to promote T-cell proliferation in response to IL-2R activation. Here, zinc causes its effect through the ERK pathway, possibly by inhibiting the dephosphorylation of MEK and ERK [37]. Additionally, zinc regulates inflammatory signaling in monocytes treated with lipopolysaccharide (LPS), interacting with cyclic nucleotide phosphodiesterases and MAPK phosphatases [38-40]. NFkB is a transcription factor involved in cellular responses to stressful stimuli including cytokines, free radicals, ultraviolet irradiation, oxidized LDL, and bacterial or viral infection that plays a key role in regulating the immune response [41]. Zinc regulates upstream signaling pathways leading to the activation of this transcription factor [38], as well as potentially regulating NFkB itself [42]. Interestingly, peripheral blood mononuclear cells (PBMC) from zinc-deficient elderly individuals show impaired NFkB activation and diminished interleukin (IL-2) production in response to stimulation with the mitogen phytohemagglutinin (PHA), corrected by *in vivo *and *in vitro *supplementation of zinc [43].

In studies measuring changes in intracellular ions such as calcium and magnesium, the tools used are partially sensitive to zinc as well. Accurate measurement of intracellular zinc requires indicators with high zinc selectivity. Currently, the single wavelength dye FluoZin-3 (Invitrogen) responds to small zinc loads, is insensitive to high calcium and magnesium ions, and is relatively unaffected by low pH or oxidants [44]. It is noteworthy that FluoZin-3 fluorescence is non-ratiometric and thus precludes a precise quantitative determination of labile zinc, a long sought after goal. Measuring "free zinc" is complicated by the relative abundance of unoccupied high-affinity binding sites in most cells. Correctly ascertaining free zinc would depend on several factors, including the buffering capacity and the dissociation constant of the zinc chelating agent [45,46].

## Zinc and the Immune Response

Zinc deficiency affects multiple aspects of innate and adaptive immunity, the consequences of which in humans include thymic atrophy, altered thymic hormones, lymphopenia, and compromised cellular-and antibody-mediated responses that result in increased rates and duration of infection. Zinc deficiency also plays a role in the immunosenescence of the elderly [47]. Changes in gene expression for cytokines, DNA repair enzymes, zinc transporters, and signaling molecules during zinc deficiency suggest that cells of the immune system are adapting to the stress of suboptimal zinc [48]. Furthermore, oral zinc supplementation improves immunity and efficiently down-regulates chronic inflammatory responses [34]. These general findings suggest that zinc is critical for normal immune cell function, whereby zinc depletion causes immune cell dysfunction, and zinc supplementation can either restore function in the setting of dysfunction or improve normal immune cell function [49].

## Zinc and Adaptive Immunity

The adaptive immune response is based on two groups of lymphocytes, B cells that differentiate into immunoglobulin secreting plasma cells and thereby induce humoral immunity, and T cells that mediate cytotoxic effects and helper cell functions of cell mediated immunity [34]. The known interactions of zinc and the immune system are categorized in Table 1 and Table 2. Both responses depend on the clonal expansion of cells following recognition of their cognate antigen.

**Table 1:** Zinc and Immune Cell Functions

Cell Type	Comment	References
Macrophages	MT-knockout results in defects in phagocytosis and antigen presentation	[73]

Dendritic cells	Zinc induces maturation and increases surface MHCII	[70]

NK cells	Zinc increases cytotoxicity and restores IFN-γ production	[50,52,61]

NKT cells	Zinc release from MTs in limited during chronic stress. Stress and inflammation induce MT gene expression, further sequestering zinc	[31,66,67]

iNKT cells	Cells lacking PLZF lack innate cytotoxicity and do not secrete IL-4 and IFN-γ	[68]

CD4 thymocytes	Zinc deficiency elevates glucocorticoid levels, causing apoptosis and reduced numbers of thymocytes	[52,57]

CD4 helper T cells	Zinc deficiency shifts Th1 to Th2 response via altered cytokine release	[10,48,56,176]

CD8 thymocytes	Zinc deficiency results in reduced numbers of thymocytes due glucocorticoid-induced apoptosis	[48,52]

T cells	Zinc deficiency results in decreased function due reduced biologically active thymulin	[53-55]

T reg	?	

Mast cells	Required for IL-6 and TNF-α production	[71,72]

**Table 2:** Zinc and Proteins of Immunological Significance

Protein	Immunological Role	References
Calcineurin	Zinc inhibits Calcineurin activity in Jurkat cells	[177]

COX-2	Lung zinc exposure increases COX-2	[178]

Caspases	Cytosolic caspase-3 activity is increased in Zn-deficient cells. May be mediated by the cytoprotectant abilities of zinc	[110]

E-selectin	Zinc deficiency increased E-selectin gene expression	[179]

FC epsilon RI	Mast cell activation downstream of FC epsilon requires zinc	[72,180]

HMGB1	3 Cys, 2 His, unknown role of zinc	[174]

HSP70	Zinc increased basal/stress-induced Hsp70 in CD3+ lymphocytes	[181]

IFN-γ	ZIP8 influences INF-gamma in T cells	[177]

IL-1 β	Zinc suppresses IL-1 beta expression in monocytes	[39,182]

IL-2	High zinc decreased IL-2 in T cell line, Jurkat cells	[183,184]

IL-2R α	High zinc decreased IL-2R α in T Cell Line	[184]

IL-6	Zinc modulated circulating cytokine in elderly patients	[61,185,186]

KIR	Zinc is necessary for the inhibitory function of KIRs	[187,188]

MCP-1	Zinc modulated circulating MCP-1 in elderly patients	[185]

MHC Class II	There is zinc dependent binding site where super-antigens and peptides bind	[189,190]

NFkB	NFkB p65 DNA-binding activity increased by zinc deficiency (sepsis). Zinc regulates NFkB. High zinc decreases NFkB activation in T Cell Line. Zinc activates NFkB in T cell line. IKK gamma zinc finger, can regulate NFkB	[42,179,191,192]

PDE-1,3,4	Zinc reversibly inhibited enzyme activity of phosphodiesterases.	[39]

PPAR-α	Zinc deficiency down-regulated PPAR-α	[184]

Proteasome	Zinc can inhibit proteasome	[193]

S100 Proteins	RAGE ligands	[173]

TLR-2	Zinc limits TLR surface expression	[194]

TNF-α	Zinc suppresses TNF-α expression in T-Cells, monocytes	[39,40,184]

**Zinc finger proteins**		

A20 zinc finger	Modulates TLR-4 signaling, Inhibits TNF-induced apoptosis	[192,195]

DPZF	BCL-6 Like Zinc Finger, Immune responses	[196]

Gfi1	Antagonizes NFkB p65, Upstream of TNF	[197,198]

IKK γ	Zinc finger that regulates NFkB	[199]

PLZF	Expressed in iNKT cells. iNKT cells lacking PLZF lack innate cytotoxicity and do not secrete IL-4 or IFN-γ	[68]

ZAS3	Zinc Finger protein that inhibits NFkB	[200]

Zinc deficiency adversely affects lymphocyte proliferation. Zinc deficient conditions are associated with elevated glucocorticoids, which cause thymic atrophy and accelerate apoptosis in thymocytes, thereby reducing lymphopoiesis [50,51]. In murine studies, zinc-deficient diets cause substantial reductions in the number of CD4+ and CD8+ thymocytes with the observation. Naïve cells sustain high levels of apoptosis in response to zinc-deficiency-induced elevated levels of glucocorticoids. Mature CD4+ and CD8+ T cells are resistant to zinc deficiency and can survive thymic atrophy, possibly because of higher levels of the anti-apoptotic protein BCL2 [48,52]. Interestingly, myelopoiesis is preserved in zinc deficiency, thereby sustaining some aspects of innate immunity.

Arguably the most prominent effect of zinc deficiency is a decline in T cell function that results from multiple causes. First, thymulin, a hormone secreted by thymic epithelial cells that is essential for the differentiation and function of T cells, requires zinc as a cofactor and exists in the plasma in a zinc-bound active form, and a zinc-free, inactive form [34]. In mice with normal thymic function, zinc deprivation reduces the level of biologically active thymulin in the circulation [53], thereby reducing the number of circulating T cells. Zinc supplementation reverses this effect [54,55].

Second, zinc deficiency leads to altered gene expression in T cells resulting in an imbalance between the peripheral functions of the Th1 and Th2 cell populations [10]. Zinc deficiency decreases production of the Th1 cell cytokines, IFN-γ, IL-2, and tumor necrosis factor (TNF)-α, which play major roles in tumor suppression. These in turn inhibit the functional capacity of these cells. Production of the Th2 cytokines IL-4, IL-6, and IL-10 are not affected. Regeneration of CD4+ T lymphocytes and CD8+ CD73+ CD11b-, precursors of cytolytic T cells, are decreased in zinc-deficient subjects with impaired immune function. An imbalance between Th1 and Th2 cells, decreased recruitment of T naive cells, and decreased percentage of T cytolytic cells are likely responsible for the cell-mediated immune dysfunction observed in zinc-deficient subjects [56,57].

Third, in mice, modest zinc deficiencies alter levels of specific thymic mRNA and proteins even before alterations occur in thymocyte development. Specifically, zinc deficiency depresses expression of myeloid cell leukemia sequence-1 (MCL1), the longer product enhancing cell survival while the alternatively spliced (shorter) form promoting apoptosis. It also enhances expression of the DNA damage repair and recombination protein 23B (RAD23B), and the mouse laminin receptor (LAMR1) and the lymphocyte-specific protein tyrosine kinase (LCK) [58], perhaps as secondary effects. Conversely, zinc supplementation suppresses the development of Th17 cells in both mouse models and cultured human and mouse leukocyte cell lines. *In vivo *and *in vitro*, zinc inhibits IL-6 induced phosphorylation of STAT3, and this observation could in part explain how zinc impedes the formation of a Th17 response [59].

## Role in Innate Immunity

Natural killer (NK) cells, dendritic cells (DCs), macrophages, mast cells, granulocytes, and complement components represent central elements of innate immunity. As observed in adaptive immune cell function, zinc deficiency results in immune dysfunction in innate immunity as well. Specifically, zinc deficiency reduces the lytic activity of natural killer cells, impairs NKT cell cytotoxicity and immune signaling, impacts the neuroendocrine-immune pathway, and alters cytokine production in mast cells [60-62]. Zinc supplementation enhances innate immunity against enterotoxigenic *E.coli *infection in children due to increases in C3 complement, enhanced phagocytosis, and T cell functionality [63].

### NK cells

Zinc deficiency reduces NK cell lytic activity in zinc deficient patients, while zinc supplementation improves NK cell functions. For example, zinc treatment at physiological doses for one month in elderly infected patients, increases NK cell cytotoxicity and enhances recovery of IFN-γ production leading to a 50% reduction in relapse of infection [61]. Additionally, *in vitro*, zinc supplementation improves the development of NK cells from CD34+ cell progenitors via increased expression of GATA-3 transcription factor [60]. Notably, centenarians have well-preserved NK cell cytotoxicity, zinc ion bioavailability, satisfactory IFNγ production, and preserved thyroid hormone turnover [62], suggesting the importance of zinc in maintaining both NK cell function and the immunologically involved neuroendocrine pathway in the elderly. Its role in regulating Class I MHC molecules has not been extensively studied, but it does appear that it is critical for HLA-C interaction with killer cell Ig-like receptors (KIRs). Interestingly, the kinetics of the binding of KIR to their respective individual Class I MHC ligands is altered significantly in the presence of zinc, but not other divalent cations. Zinc-induced multimerization of the KIR molecules may be critical for formation of KIR and HLA-C molecules at the interface between the NK cell and target cells [30].

Metallothioneins (MTs), small cysteine-rich proteins that bind zinc as well as other metal ions, mediate zinc homeostasis, and are therefore critical to not only NK function but also other cellular functions. Recent studies in aging show a novel polymorphism in the MT1A coding region in MT genes that affects NO-induced zinc ion release from the protein [64]. Other polymorphisms in MT genes impair innate immunity, further confirming a link among zinc, MT, and the innate immune response during aging.

### NKT Cells

NKT cells are a bridge between the innate and the adaptive immune systems [65], displaying both cytotoxic abilities as well as providing signals required for driving the adaptive immune response. Both zinc and MTs affect NKT cell development, maturation, and function. In conditions of chronic stress including aging, zinc release by MTs is limited, leading to low intracellular zinc bioavailability and subsequent reduced immunity [31]. Furthermore, during stress and inflammation, expression of MTs is induced by the pro-inflammatory cytokines IL-1, IL-6, and tumor necrosis factor (TNF)-α [66], resulting in further sequestration of zinc by MTs [67].

Additionally, some zinc finger motifs play an important role in the immune response of NKT cells. The BTB-ZF transcriptional regulator, promyelocytic leukemia zinc finger (PLZF), is specifically expressed in invariant natural killer T (iNKT) cells (Table 2). In the absence of PLZF, iNKT cells have markedly diminished innate cytotoxicity and do not secrete IL-4 or IFN-γ following activation [68]. Thus, zinc deficiency causes a reduction in both innate and adaptive immune functioning in NKT cells.

### Hormonal Influence

Hormones from the hypothalamic-pituitary-gonadal axis (i.e. FSH, ACTH, TSH, GH, T3, T4, insulin, and the sex hormones) directly affect the innate immune response, interacting with hormone receptors on immune cells, including NK cells. Hormonally activated NK cells produce cytokines that mediate adaptive immune responses. Deficient production of these hormones impairs innate and adaptive immune response in aging. The beneficial effects of hormone supplementation on immunity are mediated in part by enhanced intestinal zinc absorption. Therefore, zinc is a nutritional factor pivotal in maintaining the neuroendocrine-immune axis [69].

### Dendritic cells (DCs)

**Figure 1:**
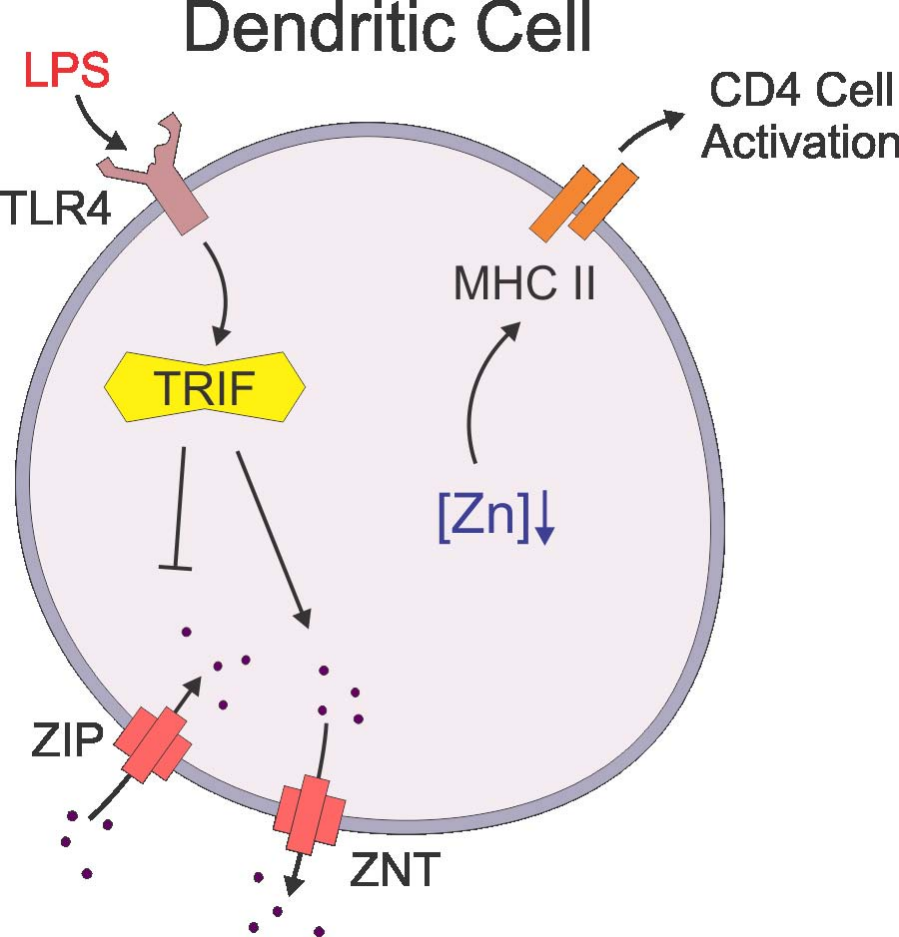
**Intracellular Zinc Levels Fall During Dendritic Cell Maturation**. After the detection of LPS (Pathogen Associated PAMPs) by TLR4 and activation of TRIF, zinc importers (ZIPs) expression is diminished while transporters (ZNTs) expression is increased. The resulting decrease in intracellular zinc concentration promotes the surface expression of MHC-II and thus the maturation of DCs.

DCs are also profoundly affected by zinc. Exposure of mouse dendritic cells to LPS, a toll-like receptor 4 (TLR4) ligand, leads to a decrease in the intracellular free zinc concentration and a subsequent increase in surface expression of MHC Class II (Figure 1), thereby enhancing DC stimulation of CD4 T cells [70]. Conversely, artificially elevating intracellular zinc levels suppresses the ability of DCs to respond to LPS. Zinc suppresses the surface expression of MHC class II molecules two ways: it inhibits the LPS-induced movement of MHC class II containing vesicles to the cell surface from the perinuclear region, and it promotes endocytosis of MHC class II molecules expressed on the plasma membrane. Zinc down-regulates the expression of the zinc importer, ZIP6 (see below), resulting in reduced intracellular zinc concentrations. Over-expression of ZIP6 suppresses DC expression of MHC class II (and subsequent stimulation of CD4+ T cells) [70]. *In vivo*, injections of LPS or a zinc chelator, N,N,N,N - tetrakis -2- pyridylmethylethylenediamine (TPEN), reduce the expression of the ZIP importers and increase the expression of zinc exporters, thereby reducing intracellular free zinc and increasing the surface expression of MHC class II. Intracellular zinc trafficking is thus important in DC maturation and subsequent T-cell activation [70]. While the observed decrease in intracellular zinc and subsequent enhancement of DC immune signaling may seem contrary to that observed with other immune cells, it should be noted that DCs undergo apoptosis following activation of their lymphocyte target(s) in the secondary lymph node sites. Therefore, upregulated immune signaling via MHCII is an effect that is followed by cell death, which is congruent with the effects of zinc depletion observed in other immune cell types.

### Mast Cells

In mast cells, an increase in intracellular free zinc, known as the 'zinc wave', occurs within minutes of extracellular stimulation [71]. This rapid response in mast cells is in contrast to changes observed in intracellular zinc in DCs, which are dependent on transcriptional regulation in zinc transporters and are therefore observed several hours following stimulation. Zinc deficiency in mast cells prevents translocation of PKC and downstream events such as the phosphorylation and nuclear translocation of NFκB as well as the downstream production of the cytokines IL-6 and TNFα [72]. Additionally, the granules of mast cells (and other immune cells) have high concentrations of zinc, which upon release could alter the extracellular milieu as well as immune, stromal, and epithelial/tumor cell functions.

### Macrophages

Macrophages from metallothionein knockout (MT-KO) mice have defects in phagocytosis, cytokine production, and antigen presentation [73]. Production of IL-1., IL-6, IL-10, and IL-12 as well as the expression of CD80, CD86 and MHC Class II molecules are reduced in macrophages from MT-KO mice. Therefore, zinc regulation by MTs plays an important role in the regulation of macrophage immune function. In some studies, zinc supplementation of human PBMCs increases mRNA production and subsequent release of the cytokines IL-6, IL-1β, and TNF-α [74], promoting the recruitment of leukocytes to the site of infection [34]. Conversely, zinc treatment suppresses the formation of pro-inflammatory cytokines [75,76]. It is thought that the effect of zinc is concentration dependent, and that zinc can be either stimulatory or inhibitory: an increase of intracellular free zinc induces cytokine production of monocytes in response to LPS [40], while higher concentrations can have the opposite effect by inhibiting cyclic nucleotide phosphodiesterases and subsequently activating protein kinase A [34,39]. Zinc can also suppress monocyte LPS-induced tumor necrosis factor (TNF)-α and IL-1β release, through inhibition of phosphodiesteras-mediated hydrolysis of cyclic nucleotides into 5′-nucleotide monophosphate and increases of intracellular cGMP levels. The NO donor s-nitroso-cysteine (SNOC) also inhibits LPS-induced TNF-α and IL-1β release, and increased levels of intracellular free zinc [77].

### Parenchymal Cells

Zinc has also been shown to be important regulators of immunity through its impact on non-circulating cells. Zinc deficiency promotes sepsis invoked organ damaged due to its effects in the epithelial cells of most organs [78]. In the lung parenchyma for example, zinc can act to diminish inflammation, and promote cell health and survival [79].

## Role in Oncogenesis

Zinc helps to maintain intracellular ion homeostasis and contributes to signal transduction in most cells. As such, zinc directly affects tumor cells through its regulatory role in gene expression and cell survival, both of which are controlled at least in part by tumor-induced alterations in zinc transporter expression, and influences tumor cells indirectly by affecting the activation, function, and/or survival of immune cells [77].

Levels of zinc in serum and malignant tissues of patients with various types of cancer are abnormal, supporting the involvement of zinc in cancer development. Studies of the role of zinc in malignant diseases have a long history of contradictory and ill-defined biological effects [80]. It is clear, however, that serum zinc levels are reduced in patients with cancers of the breast [81], gallbladder [82], lung [83], colon, head and neck [84] and bronchus [83,85,86], and in the leukocytes and granulocytes of patients with bronchus and colon cancer [86]. Serum and tumor zinc levels in human cancer are summarized in Table 3. Interestingly, while serum zinc levels are low in the setting of most cancers, tumor tissue in breast and lung cancer have elevated zinc levels when compared with the corresponding normal tissues [86,87]. Additionally, peripheral tissue surrounding liver, kidney, and lung metastasis have higher zinc content than the corresponding normal tissue or the tumor tissue itself [86]. While data of zinc levels in tumor tissue is limited, it has been widely recognized that ZIP, cellular zinc importers, are upregulated in most cancers (see below and Table 4), thereby indicating increased zinc concentrations in most tumor.

**Table 3:** Zinc Levels in Tumor Tissue

Cancer	Zinc level	References
Breast, gallbladder, colon, bronchus, lung	Decreased serum zinc	[81-83,86]

Liver, kidney, lung	Increased zinc in peritumor tissue as compared to both normal tissue and tumor itself	[86]

Breast, lung (likely others except prostate)	Increased zinc in tumor tissue	[86,87]

Prostate	Decreased zinc in tumor tissue	[86,88]

Head and Neck	Increasing zinc improves local free survival, Decreased serum zinc near end of life	[84,201]

**Table 4:** Zinc Transporters (Importers) and Cancer

Cancer	Transporter	Comment	References
Erythroleukemia	ZIP1	In the vesicular compartment and partly in the ER in adherent cells	[99]

Squamous cell carcinoma	ZIP2	mRNA is induced by contact inhibition and serum starvation	[202]

Prostate	ZIP1, ZIP2, ZIP3	Down-regulated in malignant cells	[203]

Pancreas	ZIP4	Over-expression is linked to increased cell proliferation	[106]

Breast	ZIP6, ZIP10	Expression is linked to metastasis to lymph node	[204,205]

Tamoxifen resistant breast cancer	ZIP7	Increased levels results in increased growth and invasion	[182,206,207]

Prostate tumor cells and skin cancer are the exception to these findings, in that zinc levels are lower in prostate tumor tissue than in normal prostate cancer [86,88]. Prostate glandular epithelium has the specialized function of producing and secreting large quantities of citrate, and thus requires metabolic activities that are unique to these cells. Zinc accumulation in these cells is critical to their specialized metabolism. In malignant prostate cells, the normal zinc-accumulating epithelial cells undergo a metabolic transformation causing them to lose the ability to accumulate zinc. Genetic alteration in the expression of the ZIP1 zinc importer is associated with a metabolic transformation analogous to the changes observed in malignant prostate. In fact, ZIP1, ZIP2, and ZIP3 are down-regulated in prostate cancer cells, suggesting that changes in intracellular zinc play a role in tumorigenesis. In a study by Gonzalez et al. [89], dietary zinc was not associated overall risk of prostate cancer, but long-term supplemental zinc intake was associated with reduced risk of advanced prostate cancer. Authors note much variability in current studies correlating zinc and prostate cancer. High extracellular zinc is also important, since it was shown to induce cytotoxicity in human pancreatic adenocarcinoma cell lines. Normal human pancreatic islet cells tolerated high zinc, making zinc elevation a potential treatment avenue [90]. Zinc could prevent UVB-induced aging and skin cancer development through the induction of HIF-1alpha, a protein that controls the keratinocyte cell cycle, and is down-regulated by UVB and therefore involved in UVB-induced skin hyperplasia [91].

HDAC inhibitors are being used as anticancer agents given their wide range of substrates, including proteins that have roles in gene expression, cell proliferation, cell migration, cell death, immune pathways, and angiogenesis. There are eleven **zinc dependent **HDACs in humans. The synergy of HDAC is with current anti-cancer therapies including radiation, anti-metabolites, anti-microtubule agents, topoisomerase inhibitors, DNA cross-linking agents, monoclonal antibodies, and EFGR inhibitors have been the topic of many studies [92]. Other zinc-finger transcription factors may directly influence tumor formation through the epithelial-mesenchymal transition. SNAIL, MUC1, ZEB1 are known to influence the transition away from non-tumorous epithelial lineages back to the more invasive lineages, and are effected by zinc changes [93-95].

Zinc levels are directly affected by the tumor microenvironment. Pro-inflammatory mast cells are found within the cancer microenvironment and release granules with high levels of zinc into the surrounding tissue [77]. Mast cell presence within tumors is thought to worsen the prognosis of most patients with cancer, and changes in extracellular zinc affect the cellular response in the tumor environment. Many cytokines and growth factors produced in the tumor microenvironment, including IL-6, hepatocyte growth factor, epidermal growth factor, and TNF-α, directly or indirectly affect the expression of various zinc transporters [96], thereby changing the intracellular concentrations of zinc in both tumor cells and neighboring tissues (see following section). Furthermore, it is likely that the activities of many enzymes and transcription factors that require zinc to function are affected by the altered zinc concentrations found within the cancer microenvironment. Oxidation/reduction reactions in tumors and surrounding tissues influence intracellular free zinc concentrations [77] and indeed, zinc levels may be an early intracellular 'reporter' of reactive oxygen species and subsequent biologic responses.

## Zinc Transport and Cancer

**Figure 2:**
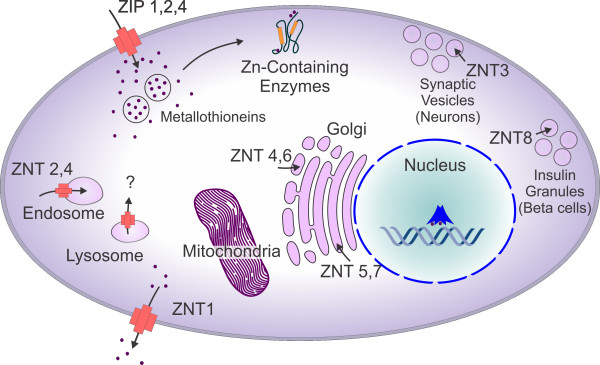
**Localization and transport of zinc in a mammalian cell**. Cellular localization and function of ZIP and ZNT zinc transporter family members. Arrows indicate the direction of zinc mobilization. ZIP1, 2 and 4 are induced in zinc deficient conditions, while ZNT-1 and 2 members are induced by zinc administration. In general zinc efflux is associated with enhanced susceptibility to apoptosis and higher levels with protection/autophagy.

Eukaryotic cells have a remarkable ability to regulate the levels of intracellular zinc. Although zinc is commonly reported to be femtomolar in concentration, it is actually found in high picomolar ranges in eukaryotic cells [45,46,97]. Several proteins, including the ZIP (ZRT-and IRT-like proteins (SLC39A)), ZNT (Zinc transporter (SLC30A)), and zinc-sequestering MTs, maintain intracellular zinc homeostasis [98-101]. ZIP members facilitate zinc influx into the cytosol from extracellular fluid or from intracellular vesicles, while ZNT proteins lower intracellular zinc by mediating zinc efflux from the cell or influx into intracellular vesicles [98,100]. Zinc sequestration is regulated primarily through zinc-dependent control of transcription, translation, and intracellular trafficking of transporters [101,102]. Expression levels of zinc transporters in human tumors correlate with their malignancy, suggesting that alteration of intracellular zinc homeostasis can contribute to the severity of cancer [103-106]. There are at least 14 human ZIP transporters, which allow zinc influx into the cell [107,108]. Specific zinc importers are upregulated in most cancer types, perhaps allowing tumor cells to escape apoptosis and activate cell survival via autophagic processes. Some important zinc transporters (ZIPs and ZNTs) are shown in Table 4 and Figure 2.

## Cell Death

Apoptosis is an active, gene-directed, tightly-regulated process of programmed cell death that involves a series of cytoskeletal, membrane, nuclear, and cytoplasmic changes that culminate in condensation and fragmentation of the cell into apoptotic bodies, which are eventually cleared by phagocytosis [109]. Apoptosis is the major mechanism of cell death in the body, enabling the removal of excess, mutant, or damaged cells. In contrast to necrosis, apoptosis deletes cells without release of their contents that would otherwise provoke and possibly damage neighboring cells and result in an inflammatory response. Apoptosis consumes energy, and involves signaling pathways originating from the plasma membrane (TNF receptor family molecules including the Fas receptor ligation or lipid peroxidation), the nucleus (DNA damage/mutation) or the cytoskeleton (disruption of microtubules) [110].

The mitochondrion has a major role in the induction, regulation, and execution of apoptosis. Mitochondria coordinate apoptosis by channeling various input signals into a central pathway, which is governed by mitochondrial-associated anti-apoptotic (Bcl-2) and pro-apoptotic (Bax) families of regulators and by providing an environment for the proteolytic events that trigger processing and activation of various members of the caspase enzyme family [111]. Action of the caspases leads to morphological changes such as cell shrinkage, condensation and fragmentation of both the cytoplasm and nucleus and formation of membrane-enclosed apoptotic bodies [111,112].

Apoptosis is tightly regulated and its deregulation is central to the pathogenesis of a number of diseases--increased in neurodegenerative disorders, AIDS, and diabetes mellitus, and decreased in autoimmune disease and neoplastic malignancies [113,114]. As such, the factors that regulate the execution phases of apoptosis are of great interest as potential therapies. One of these regulators is zinc.

## Zinc and Apoptosis

At the beginning of this decade Truong-Tran et al. assembled a core picture of zinc's role in apoptosis [109]. In this picture, the presence of zinc is anti-apoptotic, and this apoptotic effect has two aspects. Firstly, zinc may directly protect cells against oxidative damage. An example of this mechanism would be the thiolate complexes that zinc forms with sulfhydryl groups in proteins. This complex is strong enough to protect and prevent protein oxidation by ROS, but is still reversible. Secondly, evidence suggested that zinc might inhibit caspase-3 activation, perhaps, again, through forming a complex with a sulfhydryl group, in this case preventing proteolysis. There have also been some studies which imply the contrary, due to zinc's ability to inhibit important ROS-protective enzymes [115,116]. In mouse DCs, zinc induces apoptosis by stimulating the formation of ceramide [117]. Similar events are observed in erythrocytes, where zinc induces secretory sphingomylenase, which produces ceramide leading to apoptosis [118].

Although high concentrations of zinc may trigger cell death by apoptosis or necrosis [119-122]in many settings, zinc is a physiological suppressor of apoptosis. There are two major anti-apoptotic mechanisms of zinc: it directly influences apoptotic regulators, especially the caspase family of enzymes, and it may prevent oxidative damage and damage induced by toxins, thereby suppressing the caspase activating pathways and apoptosis. These two mechanisms are closely related since a decline in intracellular zinc below a critical level may not only trigger pathways leading to caspase activation via increased oxidative stress, but may also directly facilitate the process by which the caspases are activated [109].

Zinc deficiency-induced apoptosis in vitro and in vivo displays all of the fundamental characteristics of apoptosis, including DNA and nuclear fragmentation, chromatin condensation and apoptotic body formation [123], indicating that apoptosis is directly related to the decrease in intracellular zinc. Zinc deficiency decreases cell proliferation and increases apoptosis in neuroblastoma IMR-32 cells. In these cells, low zinc arrests the cell cycle at G0/G1 phase, and induces apoptosis through the intrinsic pathway [124]. Specifically, cytosolic caspase-3 activity is increased in zinc deficient cells, and zinc suppresses caspase-3 activity and apoptosis in rats in vivo [125]. Taken together, this demonstrates that zinc deficiency-induced apoptosis is dependent on caspase-3 activation. Interestingly, in zinc deficiency, the frequency of apoptotic cells is significantly increased in specific tissues, including the intestinal and retinal pigmented epithelium, skin, thymic lymphocytes, testis and pancreatic acinar cells [126,127] and neuroepithelium [128]. The importance of these observed localizations has yet to be elucidated.

In 2010, our understanding of the role of zinc has progressed to the point where we understand zinc's role in apoptosis to involve both direct effects on mitochondria and the nucleus as well as on various factors and signaling pathways within and between the cytosol, mitochondria, and nucleus. We also know that within some cell types including neurons, glial cells, and prostate epithelial cells, zinc may be pro-apoptotic [129]. Still, many of the precise mechanisms through which zinc regulates apoptosis and proliferation remain to be elucidated. Interestingly a pro-apoptotic compound which increases the conversion of pro-caspase 3 to the active caspase 3 form was found to operate through the sequestration of the zinc that inhibits cleavage of the pro-caspase 3 [130].

Many animal studies have linked zinc deficiency with enhanced rates of oxidative damage [131-133]. Zinc supplementation also protects against intracellular oxidative damage. Zinc depletion increases the rate of apoptosis, and there is a synergy in the induction of apoptosis between zinc depletion and other apoptotic inducers such as colchicine, tumor necrosis factor and HIV-1 Tat protein [134,135]. Therefore, major reductions in intracellular zinc can directly induce apoptosis, while smaller decreases may increase cell susceptibility to apoptosis by other toxins.

Zinc is a cytoprotectant, and as such it protects and stabilizes proteins, DNA, cytoskeleton, organelles, and membranes [136], reminiscent of survival factors associated with autophagy. For instance, axons and dendrites exposed to zinc chelators (TPEN and zinquin) slowly "die back", due to metabolic lack of neuronal ATP, which can be resolved with addition of NAD [137]. Zinc can also up-regulate MT, which stabilize lysosomes and decrease apoptosis resulting from oxidative stress, due to increases in autophagy [138]. Cytoprotective zinc is most likely the exchangeable (loosely bound or tightly bound but kinetically labile) zinc pools [97,134,136]. Zinc protects sulfhydryl groups in proteins from oxidation by forming strong, reversible, thiolate complexes, and as such provides protection to enzymes with essential thiols such as tubulin, where sulfhydryls are required for polymerization into microtubules [139,140]. As such, zinc is a stabilizer of microtubules, and microtubule disruption occurs in zinc deficiency [141], oxidative stress [142] and in the early stages of apoptosis [143]. It is also important to note that TPEN itself or TPEN-Zinc complexes may actually be the cause of increased apoptosis in some of these experiments [144].

Supplementing cells with exogenous zinc in vitro decreases the susceptibility of cells and tissues to spontaneous or toxin-induced apoptosis. In several studies, zinc-supplemented animals have increased resistance to apoptotic inducers. For example, zinc has protective effects against whole body irradiation in mice [145], neuronal apoptosis following transient forebrain ischemia in the hippocampus of primates [146], and apoptosis of the anterior and stromal keratinocytes in the eye following superficial keratectomy in rabbits [147]. PBLs pretreated with zinc are resistant to Cr(III)(phe)3 induced apoptosis. This reduced apoptosis correlated with decreased ROS production in cells pretreated with zinc [148]. Zinc blocks apoptosis induced by all apoptosis-inducing treatments tested, indicating that it suppresses a central pathway [127,135,149]. Monocytes in chronic HIV viremia are resistant to apoptosis. Expression of MTs, which are highly involved in cellular zinc metabolism, and ZIP8 zinc importer are up-regulated in these monocytes. Increased intracellular zinc, therefore, may play a role in the apoptotic resistance seen in monocytes during HIV viremia [150].

There are several issues, however, with zinc supplementation studies and their interpretation. There is relatively poor uptake of ionic zinc across the plasma cell membrane, and mM concentrations of zinc can cross-link proteins nonspecifically, rendering interpretation difficult. Exogenous zinc driven into cells with an ionophore, such as pyrithione, has resolved many of the zinc uptake issues, but presents a secondary problem. Many zinc ionophores act on other cellular cations such as calcium and magnesium [151]. Additionally, using ionophores may produce much higher intracellular zinc levels than would occur in vivo. Metabolically available zinc is distributed non-uniformly throughout the cell with nM-pM concentrations in the cytosol and up to mM concentrations within vesicles [97]. It is unknown whether zinc supplementation affects the same pools and apoptotic targets as does zinc depletion.

## Zinc, Apoptosis and Cancer

### Role in Necrosis

In some cells, zinc deprivation results in necrosis. The reason for this has not yet been elucidated, but may depend on the functional state of activated caspases. In TPEN-induced zinc-deficient human renal cell carcinoma cell lines lacking caspases-3, -7, -8 and -10 died by necrosis rather than apoptosis [152]. In these cases, zinc may not regulate apoptosis, but rather function as a cytoprotectant that, in zinc-deficient conditions, leaves the cell vulnerable to apoptosis and necrosis.

### Zinc and Autophagy

Normal cellular growth and development require a balance between protein synthesis and degradation. Eukaryotic cells have two major avenues for degradation: the proteasome and autophagy [153]. Autophagy, literally 'self-eating', is involved in the bulk degradation of long-lived cytosolic proteins and organelles, whereas the ubiquitin-proteasome system degrades specific short-lived proteins. Autophagy is a highly conserved process in eukaryotes in which excess or aberrant organelles and their surrounding cytoplasm are sequestered into double-membrane vesicles and delivered to the lysosome for breakdown and eventual recycling of the resulting macromolecules. There are three types of autophagy, the first of which, chaperone-mediated autophagy, is a mechanism that allows the degradation of cytosolic proteins that contain a particular pentapeptide consensus motif [154,155]. The two other types of autophagy, macro-autophagy and microautophagy, involve dynamic membrane rearrangements and terminate at the lysosome [156,157] with fusion and degradation. Microautophagy is a direct engulfment of cytoplasm at the surface of the degradative organelle by protrusion, septation, and/or invagination of the membrane, while macroautophagy involves sequestering cytoplasm into a double-membrane cytosolic vesicle, the autophagosome [153]. Autophagosomes fuse with the lysosome, the contents are degraded, and the macromolecules recycled.

Autophagy has an important role in various biological events such as adaptation to changing environmental conditions [158,159], cellular remodeling during development and differentiation, and determination of lifespan [160]. Autophagy may play a protective role against the progression of some human diseases, including cancer, muscular disorders, and neurodegeneration, such as Huntington's, Alzheimer's, and Parkinson's diseases [160-162], and acts as a cellular defense mechanism to prevent infection by certain pathogenic bacteria and viruses [162-164]. Autophagy is involved in some forms of cell death and might contribute to the pathology of associated diseases [157,165].

Endogenous zinc levels appear to be critical to induce autophagy under conditions of oxidative stress in astrocytes. Autophagy is a necessary preceding event for lysosomal membrane permeabilization and cell death in oxidative injury [166]. When autophagy is induced in astrocytes, the number of autophagic vacuoles positive for LC3 (microtubule-associated protein 1 light chain 3), a marker of autophagy, increases, and levels of labile zinc increase in autophagic vacuoles as well as in the cytosol and nuclei. Interestingly, chelation of zinc with TPEN decreases the number of autophagic vacuoles in autophagy-induced astrocytes, similar to the effects observed with autophagy inhibitors (3-methyladenine, bafilomycin-1). Conversely, exposure to zinc increases the number of autophagic vacuoles. Taken together, these findings suggest that zinc is critical to autophagy. Possibly related to zinc's role in autophagy, ethambutol, an anti-tuberculosis agent, can cause irreversible vision loss, associated with severe vacuole formation in cultured retinal cells. In ethambutol-treated cultured retinal cells, almost all ethambutol-induced vacuoles contained high levels of labile zinc. Intracellular zinc chelation with TPEN blocks both vacuole formation and zinc accumulation in the vacuole, and inhibits lysosomal activation and lysosomal membrane permeabilization [167]. Although there are examples of zinc's effect on autophagy in bacteria and yeast [168], it is not as clear how these can be translated to mammals. Zn mediates tamoxifen-induced autophagy in breast cancer cells [169], hippocampal neurons [170], retinal cells [167], and in astrocytes via increases in oxidative stress and induction of lysosomal membrane permeabilization [171]. The newer studies have used animals deficient in metallothionein to study the changes and importance of zinc. Again, autophagy is now seen as a mechanism that tumor cells use to promote their survival, even in face of potent chemotherapies [169].

The alterations of free zinc concentration and zinc transporters in maturing dendritic cells suggest another, as yet unexplored intersection between zinc regulation and autophagy. After all, the activation of autophagy mechanisms is a second defining feature of DC maturation and effective MHC-II antigen loading [172].

## Summary

Significant disorders of great public health interest are associated with zinc deficiency. The amelioration of a number of common conditions with zinc supplementation in the context of malnutrition has underscored the importance of this micronutrient. Rapid advances in molecular biology and genetics have revealed the complexities in zinc homeostasis and the attendant pathophysiology of mutations in critical genes affecting usually well controlled intra-and extracellular levels of zinc. It is apparent that a labile pool of zinc contributes to a myriad of cell signaling processes providing critical insight into the role of zinc in health and disease. In the immune system, we now know that this pool can affect function, differentiation, maturation and cell death pathways in critical immunocytes thereby contributing to many aspects of innate and adaptive immunity. Similar observations are apparent in tumor cells and the critical contribution of immune cells in the microenvironment and pathogenesis of cancer underscores the potential connection between zinc homeostasis and oncology. Manipulating zinc levels in adoptively transferred immune cells thus may be an interesting and important means to alter their function, and promote either tolerance or immunity. Though biologically significant, exogenous zinc may be too blunt a tool for targeting some zinc dependent cellular processes. Drugs and treatments capable of targeting zinc levels of specific pools within the cell or that inhibit zinc binding to a restricted class of protein, may be more effective in this regard.

Among the critical limitations in advancing our understanding of the role of zinc in tumor immunology are: a) availability of quantitative zinc sensors (e.g. ratiometric fluorophores, genetically encoded and easily used detectors, etc) for cellular and organ physiology; b) improved analytical tools to approach the zinc proteome in earnest and in a more high throughput conducive fashion; c) needed progress in biomarkers of zinc deficiency and/or imaging of zinc in medicine in addition to current rather difficult to interpret measurements of total zinc in various biological compartments; d) more complete information on polymorphisms in various zinc transporters, importers and binding proteins; and e) methods of targeting specific subcellular pools of zinc. It is quite likely that alterations in zinc homeostasis may be a contributing factor in genetic alternations (ZNT, ZIP, metallothionein, etc) or environmental causes (nutritional status, exposure to zinc, microbial control) playing a role in the genesis and/or maintenance of cancer. Its role in HMGB1 and RAGE signaling in cancer has not been fully explored [173-175]. As such, a rational approach towards zinc supplementation and modulation may ultimately emerge in the context of preventing or treating immunologic and oncologic disorders.

## Competing interests

The authors declare that they have no competing interests.

## Authors' contributions

EJ was the primary writer of the review, TL wrote several sections of the review and revisions, WB wrote several sections of the review and revisions, BP, PB, LB, PK reviewed the manuscript and ML conceived of the document and drafted parts of the original document. All authors have read and approved the final manuscript.

## References

[B1] Rink L Gabriel P Zinc and the immune system Proc Nutr Soc 2000 541 10.1017/S0029665100000781 11115789

[B2] Wapnir AR Protein Nutrition and Mineral Absorption 1990 CRC Press, Boca Raton

[B3] Berdanier DC Dwyer JT Feldman EB Handbook of Nutrition and Food 2007 CRC Pres, Boca Raton

[B4] Raulin J Chemical studies on vegetation Annales des Sci Naturelles 1869 11 93 99

[B5] Sommer AL Lipman CB Evidence on indispensable nature of zinc and boron for higher green plants Plant Physiol 1926 1 231 10.1104/pp.1.3.231 16652481 PMC439917

[B6] Todd WR Elvehjem CA Hart EB Zinc in the nutrition of the rat Am J Physiol 1933 107 146 156

[B7] Follis RH Day HG McCollum EV Histological studies of the tissues of rats fed a diet extremely low in zinc J Nutr 1941 22 223

[B8] Tucker HF Salmon WD Parakeratosis or zinc deficiency disease in the pig Proc Soc Exp Biol 1955 88 613 14371717 10.3181/00379727-88-21670

[B9] Blencowe DK Morby AP Zn(II) metabolism in prokaryotes FEMS Microbiol Rev 2003 27 291 311 10.1016/S0168-6445(03)00041-X 12829272

[B10] Prasad AS Zinc: an overview Nutrition 1995 11 93 99 7749260

[B11] Prasad AS Zinc in human health: an update J Trace Elements Exp Med 1998 11 63 87 10.1002/(SICI)1520-670X(1998)11:2/3<63::AID-JTRA2>3.0.CO;2-5

[B12] Joazeiro CA Weissman AM RING finger proteins: mediators of ubiquitin ligase activity Cell 2000 102 549 552 10.1016/S0092-8674(00)00077-5 11007473

[B13] Kadrmas JL Beckerle MC The LIM domains: from the cytoskeleton to the nucleus Nat Rev Mol Cell Biol 2004 5 920 931 10.1038/nrm1499 15520811

[B14] Vallee BL The function of metallothionein Neurochem Int 1995 27 23 33 10.1016/0197-0186(94)00165-Q 7655345

[B15] National Institutes of Health, Office of Dietary Supplements Zinc: Health Professional Fact Sheet http://ods.od.nih.gov/FactSheets/Zinc.asp

[B16] Institute of Medicine, Food and Nutrition Board Dietary Reference Intakes for Vitamin A, Vitamin K, Arsenic, Boron, Chromium, Copper, Iodine, Iron, Manganese, Molybdenum, Nickel, Silicon, Vanadium, and Zinc 2001 Washington, DC: National Academy Press 25057538

[B17] Gropper SS Smith JL Groff JL Advanced nutrition and human metabolism 2009 Belmont, CA: Wadsworth

[B18] Brown H Peerson JM Allen LH Rivera J Effect of supplemental zinc on the growth and serum zinc concentrations of pre-pubertal children: a metaanalysis of randomized, controlled trials Am J Clin Nutrition 2002 75 1062 1071 10.1093/ajcn/75.6.1062 12036814

[B19] Prasad AS Halsted JA Nadimi M Syndrome of iron deficiency anemia, hepatosplenomegaly, hypogonadism, dwarfism and geophagia Am J Med 1961 31 532 546 10.1016/0002-9343(61)90137-1 14488490

[B20] Bhutta ZA Bird SM Black RE Therapeutic effects of oral zinc in acute and persistent diarrhea in children in developing countries: pooled analysis of randomized controlled trials Am J Clin Nutr 2000 72 1516 1522 11101480 10.1093/ajcn/72.6.1516

[B21] Dutta SK Procaccino F Aamodt R Zinc metabolism in patients withexocrine pancreatic insufficiency J Am Coll Nutr 1998 17 556 563 9853534 10.1080/07315724.1998.10718803

[B22] Fraker PJ King LE Laakko T Vollmer TL The dynamic link between the integrity of the immune system and zinc status J Nutr 2000 130 1399 1406 10.1093/jn/130.5.1399S 10801951

[B23] Prasad AS Clinical and biochemical manifestation zinc deficiency in human subjects J Pharmacol 1985 16 344 352 2419703

[B24] Tapazoglou E Prasad AS Hill G Brewer GJ Kaplan J Decreased natural killer cell activity in patients with zinc deficiency with sickle cell disease J Laboratory Clin Med 1985 105 19 22 3968462

[B25] Zemel BS Kawchak DA Fung EB Ohene-Frempong K Stallings VA Effect of zinc supplementation on growth and body composition in childrenwith sickle cell disease Am J Clin Nutr 2002 75 300 307 11815322 10.1093/ajcn/75.2.300

[B26] Chue CD Rajpar SF Bhat J An acrodermatitis enteropathica-like eruption secondary to acquired zinc deficiency in an exclusively breast-fed premature infant Int J Dermatol 2008 47 4 372 3 10.1111/j.1365-4632.2008.03492.x 18377601

[B27] Mocchegiani E Costarelli L Giacconi R Cipriano C Muti E Malavolta M Zinc-binding proteins (metallothionein and alpha-2 macroglobulin) and immunosenescence Exp Gerontol 2006 41 1094 1107 10.1016/j.exger.2006.08.010 17030107

[B28] Roth DE Richard SA Black RE Zinc supplementation for the prevention of acute lower respiratory infection in children in developing countries: meta-analysis and meta-regression of randomized trials Int J Epidemiol 2010 39 3 795 808 10.1093/ije/dyp391 20156999

[B29] Maret W Sandstead HH Zinc requirements and the risks and benefits of zinc supplementation J Trace Elem Med Biol 2006 20 1 3 18 10.1016/j.jtemb.2006.01.006 16632171

[B30] Vales-Gomez M Erskine RA Deacon MP Strominger JL Reyburn HT The role of zinc in the binding of killer cell Ig-like receptors to class I MHC proteins Immunology 2000 96 1734 1739 10.1073/pnas.041618298 PMC29326 11172020

[B31] Walker CL Black RE Zinc for the treatment of diarrhoea: effect on diarrhoea morbidity, mortality and incidence of future episodes Int J Epidemiol 2010 39 Suppl 1 63 9 10.1093/ije/dyq023 PMC2845862 20348128

[B32] Li Y Hough CJ Suh SW Sarvey JM Frederickson CJ Rapid translocation of Zn (2+) from presynaptic terminals into postsynaptic hippocampal neurons after physiological stimulation J Neurophysiol 2001 86 2597 2604 11698545 10.1152/jn.2001.86.5.2597

[B33] Csermely P Somogyi J Zinc as a possible mediator of signal transduction in T lymphocytes Acta Physiol Hung 1989 74 195 199 2603734

[B34] Haase H Rink L The immune system and the impact of zinc during aging Immun Ageing 2009 6 9 10.1186/1742-4933-6-9 19523191 PMC2702361

[B35] Romir J Lilie H Egerer-Sieber C Bauer F Sticht H Muller YA Crystal structure analysis and solution studies of human Lck-SH3; zinc-induced homodimerization competes with the binding of proline-rich motifs J Mol Biol 2007 365 1417 1428 10.1016/j.jmb.2006.10.058 17118402

[B36] Lefebvre DC Lai JC Maeshima N Ford JL Wong AS Cross JL Johnson P CD44 interacts directly with Lck in a zinc-dependent manner Mol Immunol 2010 47 10 1882 9 10.1016/j.molimm.2010.03.018 20417561

[B37] Kaltenberg J Plum LM Ober-Blöbaum JL Hönscheid A Rink L Haase H Zinc signals promote IL-2-dependent proliferation of T cells Eur J Immunol 2010 40 5 1496 503 10.1002/eji.200939574 20201035

[B38] Haase H Ober-Blobaum JL Engelhardt G Hebel S Heit A Heine H Rink L Zinc signals are essential for lipopolysaccharide-induced signal transduction in monocytes J Immunol 2008 181 6491 6502 18941240 10.4049/jimmunol.181.9.6491

[B39] von Bulow V Rink L Haase H Zinc-mediated inhibition of cyclic nucleotide phosphodiesterase activity and expression suppresses TNF-alpha and IL-1 beta production in monocytes by elevation of guanosine 3',5'-cyclic monophosphate J Immunol 2005 175 4697 4705 16177117 10.4049/jimmunol.175.7.4697

[B40] von Bulow V Dubben S Engelhardt G Hebel S Plumakers B Heine H Rink L Haase H Zinc-dependent suppression of TNF-alpha production is mediated by protein kinase A-induced inhibition of Raf-1, I kappa B kinase beta, and NF-kappa B J Immunol 2007 179 4180 4186 17785857 10.4049/jimmunol.179.6.4180

[B41] Gilmore TD Introduction to NF-kB: players, pathways, perspectives Oncogene 2006 25 6680 6684 10.1038/sj.onc.1209954 17072321

[B42] Bao S Liu MJ Lee B Besecker B Lai JP Guttridge DC Knoell DL Zinc modulates the innate immune response in vivo to polymicrobial sepsis through regulation of NF-kappaB Am J Physiol Lung Cell Mol Physiol 2010 298 6 L744 54 10.1152/ajplung.00368.2009 20207754 PMC2886607

[B43] Prasad AS Bao B Beck FW Sarkar FH Correction of interleukin-2 gene expression by in vitro zinc addition to mononuclear cells from zinc-deficient human subjects: a specific test for zinc deficiency in humans Transl Res 2006 148 325 333 10.1016/j.trsl.2006.07.008 17162254

[B44] Devinney MJ Reynolds IJ Dineley KE Simultaneous detection of intracellular free calcium and zinc using fura-2FF and FluoZin-3 Cell Calcium 2005 37 225 232 10.1016/j.ceca.2004.10.003 15670869

[B45] Bozym R Hurst TK Westerberg N Stoddard A Fierke CA Frederickson CJ Thompson RB Determination of zinc using carbonic anhydrase-based fluorescence biosensors Methods Enzymol 2008 450 287 309 full_text 19152866 10.1016/S0076-6879(08)03414-9

[B46] Krezel A Maret W Zinc-buffering capacity of a eukaryotic cell at physiological pZn J Biol Inorg Chem 2006 11 8 1049 62 10.1007/s00775-006-0150-5 16924557

[B47] Haase H Rink L The immune system and the impact of zinc during aging Immun Ageing 2009 12 6 9 10.1186/1742-4933-6-9 PMC2702361 19523191

[B48] Fraker PJ King LE Reprogramming of the immune system during zinc deficiency Annu Rev Nutr 2004 24 277 298 10.1146/annurev.nutr.24.012003.132454 15189122

[B49] Prasad AS Zinc: role in immunity, oxidative stress and chronic inflammation Curr Opin Clin Nutr Metab Care 2009 12 6 646 52 10.1097/MCO.0b013e3283312956 19710611

[B50] DePasquale-Jardieu P Fraker PJ The role of corticosterone in the loss in immune function in the zinc-deficient A/J mouse J Nutr 1979 109 1847 1855 315453 10.1093/jn/109.11.1847

[B51] DePasquale-Jardieu P Fraker PJ Further characterization of the role of corticosterone in the loss of humoral immunity in zinc-deficient A/J mice as determined by adrenalectomy J Immunol 1980 124 2650 2655 6966295

[B52] King LE Osati-Ashtiani F Fraker PJ Apoptosis plays a distinct role in the loss of precursor lymphocytes during zinc deficiency in mice J Nutr 2002 132 974 979 11983824 10.1093/jn/132.5.974

[B53] Iwata T Incefy GS Tanaka T Fernandes G Menendez-Botet CJ Pih K Good RA Circulating thymic hormone levels in zinc deficiency Cell Immunol 1979 47 100 105 10.1016/0008-8749(79)90318-6 509529

[B54] Dardenne M Savino W Wade S Kaiserlian D Lemonnier D Bach JF In vivo and in vitro studies of thymulin in marginally zinc-deficient mice Eur J Immunol 1984 14 454 458 10.1002/eji.1830140513 6609827

[B55] Prasad AS Meftah S Abdallah J Kaplan J Brewer GJ Bach JF Dardenne M Serum thymulin in human zinc deficiency J Clin Invest 1988 82 1202 1210 10.1172/JCI113717 3262625 PMC442670

[B56] Beck FW Prasad AS Kaplan J Fitzgerald JT Brewer GJ Changes in cytokine production and T cell subpopulations in experimentally induced zinc-deficient humans Am J Physiol 1997 272 1272 10.1152/ajpendo.1997.272.6.E1002 9227444

[B57] Prasad AS Beck FW Grabowski SM Kaplan J Mathog RH Zinc deficiency: changes in cytokine production and T-cell subpopulations in patients with head and neck cancer and in noncancer subjects Proc Assoc Am Physicians 1997 109 68 77 9010918

[B58] Moore JB Blanchard RK McCormack WT Cousins RJ cDNA array analysis identifies thymic LCK as upregulated in moderate murine zinc deficiency before T-lymphocyte population changes J Nutr 2001 131 3189 3196 11739864 10.1093/jn/131.12.3189

[B59] Kitabayashi C Fukada T Kanamoto M Ohashi W Hojyo S Atsumi T Ueda N Azuma I Hirota H Murakami M Hirano T Zinc suppresses Th17 development via inhibition of STAT3 activation Int Immunol 2010 22 5 375 86 10.1093/intimm/dxq017 20215335

[B60] Muzzioli M Stecconi R Moresi R Provinciali M Zinc improves the development of human CD34+ cell progenitors towards NK cells and increases the expression of GATA-3 transcription factor in young and old ages Biogerontology 2009 10 5 593 604 10.1007/s10522-008-9201-3 19043799

[B61] Mocchegiani E Muzzioli M Giacconi R Cipriano C Gasparini N Franceschi C Gaettic R Cavalierid E Suzukid H Metallothioneins/PARP-1/IL-6 interplay on natural killer cell activity in elderly: parallelism with nonagenarians and old infected humans. Effect of zinc supply Mech Ageing Dev 2003 124 10.1016/s0047-6374(03)00023-x 12714254

[B62] Mariani E Ravaglia G Forti P Meneghetti A Tarozzi A Maioli F Boschi F Pratelli L Pizzoferrato A Piras F Facchini A Vitamin D, thyroid hormones and muscle mass influence natural killer (NK) innate immunity in healthy nonagenarians and centenarians Clin Exp Immunol 1999 116 19 27 10.1046/j.1365-2249.1999.00855.x 10209500 PMC1905230

[B63] Sheikh A Shamsuzzaman S Ahmad SM Nasrin D Nahar S Alam MM Al Tarique A Begum YA Qadri SS Chowdhury MI Saha A Larson CP Qadri F Zinc Influences the Innate Immune Responses in Children with Enterotoxigenic Escherichia coli-Induced Diarrhea J Nutr 2010 140 5 1049 56 10.3945/jn.109.111492 20237063

[B64] Cipriano C Malavolta M Costarelli L Giacconi R Muti E Gasparini N Cardelli M Monti D Mariani E Mocchegiani E Polymorphisms in MT1a gene coding region are associated with longevity in Italian Central female population Biogerontology 2006 7 357 365 10.1007/s10522-006-9050-x 16955215

[B65] Taniguchi M Seino K Nakayama T The NKT cell system: bridging innate and acquired immunity Nat Immunol 2003 4 1164 1165 10.1038/ni1203-1164 14639465

[B66] Davis SR Cousins RJ Metallothionein expression in animals: a physiological perspective on function J Nutr 2000 13 1085 1088 10.1093/jn/130.5.1085 10801901

[B67] Mocchegiani E Giacconi R Muti E Cipriano C Costarelli L Tesei S Zinc-bound metallothioneins and immune plasticity: lessons from very old mice and humans Immun Ageing 2007 4 1 7 10.1186/1742-4933-4-1 17903270 PMC2082024

[B68] Kovalovsky D Uche OU Eladad S Hobbs RM Yi W Alonzo E Chua K Eidson M Kim H-J Im JS Pandolfi PP Sant'Angelo DB The BTB-zinc finger transcriptional regulator, PLZF, controls the development of iNKT cell effector functions Nat Immunol 2008 9 1055 1064 10.1038/ni.1641 18660811 PMC2662733

[B69] Mocchegiani E Giacconi R Cipriano C Malavolta M NK and NKT Cells in Aging and Longevity: Role of Zinc and Metallothioneins Journal of Clinical Immunology 2009 29 416 425 10.1007/s10875-009-9298-4 19408107

[B70] Kitamura H Morikawa H Kamon H Iguchi M Hojyo S Fukada T Yamashita S Kaisho T Akira S Murakami M Hirano T Toll-like receptor-mediated regulation of zinc homeostasis influences dendritic cell function Nat Immunol 2006 7 971 977 10.1038/ni1373 16892068

[B71] Yamasaki S Sakata-Sogawa K Hasegawa A Suzuki T Kabu K Sato E Kurosaki T Yamashita S Tokunaga M Nishida K Hirano T Zinc is a novel intracellular second messenger J Cell Biol 2007 177 637 645 10.1083/jcb.200702081 17502426 PMC2064209

[B72] Kabu K Yamasaki S Kamimura D Ito Y Hasegawa A Sato E Kitamura H Nishida K Hirano T Zinc is required for Fc epsilon RI-mediated mast cell activation J Immunol 2006 177 2 1296 305 16818790 10.4049/jimmunol.177.2.1296

[B73] Sugiura T Kuroda E UY Dysfunction of macrophages in metallothioneinknock out mice J UOEH 2004 26 193 205 15244072 10.7888/juoeh.26.193

[B74] Wellinghausen N Kirchner H Rink L The immunobiology of zinc Immunol Today 1997 18 519 521 10.1016/S0167-5699(97)01146-8 9386346

[B75] Bao B Prasad AS Beck FW Godmere M Zinc modulates mRNA levels of cytokines Am J Physiol Endocrinol Metab 2003 285 E1095 1102 12812920 10.1152/ajpendo.00545.2002

[B76] Zhou Z Wang L Song Z Saari JT McClain CJ Kang YJ Abrogation of nuclear factor-kappaB activation is involved in zinc inhibition of lipopolysaccharide-induced tumor necrosis factor-alpha production and liver injury Am J Pathol 2004 164 1547 1556 15111301 10.1016/s0002-9440(10)63713-3 PMC1615672

[B77] Murakami M Hirano T Intracellular zinc homeostasis and zinc signaling Cancer Sci 2008 99 1515 1522 10.1111/j.1349-7006.2008.00854.x 18754861 PMC11158020

[B78] Knoell DL Julian MW Bao S Besecker B Macre JE Leikauf GD DiSilvestro RA Crouser ED Zinc deficiency increases organ damage and mortality in a murine model of polymicrobial sepsis Crit Care Med 2009 37 4 1380 8 10.1097/CCM.0b013e31819cefe4 19242332 PMC2905048

[B79] Zalewski PD Zinc metabolism in the airway: basic mechanisms and drug targets Curr Opin Pharmacol 2006 6 3 237 43 10.1016/j.coph.2006.01.005 16540372

[B80] Mulay IL Roy R Knox BE Suhr NH Delaney WE Trace-metal analysis of cancerous and noncancerous human tissues J Natl Cancer Inst 1971 47 1 13 4328191

[B81] Schlag P Seeling W Merkle P Betzler M Changes of serum-zinc in breast cancer Langenbecks Arch Chir 1978 2 129 133 10.1007/BF01261399 682772

[B82] Gupta SK Singh SP Shukla VK Copper, zinc, and Cu/Zn ratio in carcinoma of the gallbladder J Surg Oncol 2005 91 204 208 10.1002/jso.20306 16118778

[B83] Issell BF Macfadyen BV Gum ET Valdivieso M Dudrick SJ Bodey GP Serum zinc levels in lung cancer patients Cancer 2006 47 1845 1848 10.1002/1097-0142(19810401)47:7<1845::AID-CNCR2820470721>3.0.CO;2-B 7226079

[B84] Büntzel J Bruns F Glatzel M Garayev A Mücke R Kisters K Schäfer U Schönekaes K Micke O Zinc concentrations in serum during head and neck cancer progression Anticancer Res 2007 27 4A 1941 3 17649800

[B85] Chakravarty PK Ghosh A Chowdhury JR Zinc in human malignancies Neoplasma 1985 33 85 90 3960212

[B86] Schwartz M Role of trace elements in cancer Cancer Res 1975 35 3481 3487 1104155

[B87] Margalioth EJ Schenker JG Chevion M Copper and zinc levels in normal and malignant tissues Cancer Sci 1983 52 868 872 10.1002/1097-0142(19830901)52:5<868::aid-cncr2820520521>3.0.co;2-k 6871828

[B88] Costello LC Franklin RB The clinical relevance of the metabolism of prostate cancer; zinc and tumor suppression: connecting the dots Mol Cancer 2006 5 17 10.1186/1476-4598-5-17 16700911 PMC1481516

[B89] Gonzalez A Peters U Lampe JW White E Zinc intake from supplements and diet and prostate cancer Nutr Cancer 2009 61 2 206 15 10.1080/01635580802419749 19235036 PMC2664741

[B90] Jayaraman AK Jayaraman S Increased level of exogenous zinc induces cytotoxicity and up-regulates the expression of the ZnT-1 zinc transporter gene in pancreatic cancer cells J Nutr Biochem 2010 In Press Corrected Proof, Available online 14 April 2010 20392624 10.1016/j.jnutbio.2009.12.001

[B91] Cho YS Lee KH Park JW Pyrithione-zinc Prevents UVB-induced Epidermal Hyperplasia by Inducing HIF-1alpha Korean J Physiol Pharmacol 2010 14 2 91 7 10.4196/kjpp.2010.14.2.91 20473380 PMC2869458

[B92] Marks PA Histone deacetylase inhibitors: A chemical genetics approach to understanding cellular functions Biochim Biophys Acta 2010 In press corrected proof, Available online 8 June 2010 20594930 10.1016/j.bbagrm.2010.05.008 PMC4043339

[B93] Yamashita S Miyagi C Fukada T Kagara N Che YS Hirano T Zinc transporter LIVI controls epithelial-mesenchymal transition in zebrafish gastrula organizer Nature 2004 429 6989 298 302 10.1038/nature02545 15129296

[B94] Cano A Pérez-Moreno MA Rodrigo I Locascio A Blanco MJ del Barrio MG Portillo F Nieto MA The transcription factor Snail controls epithelial-mesenchymal transitions by repressing E-cadherin expression Nat Cell Biol 2002 2 2 76 83 10.1038/35000025 10655586

[B95] Guaita S Puig I Franci C Garrido M Dominguez D Batlle E Sancho E Dedhar S De Herreros AG Baulida J Snail induction of epithelial to mesenchymal transition in tumor cells is accompanied by MUC1 repression and ZEB1 expression J Biol Chem 2002 277 42 39209 16 10.1074/jbc.M206400200 12161443

[B96] Ibs KH Rink L Zinc-altered immune function J Nutr 2003 133 5 Suppl 1 1452 6 10.1093/jn/133.5.1452S 12730441

[B97] Frederickson CJ Neurobiology of zinc and zinc-containing neurons Int Rev Neurobiol 1989 31 145 238 full_text 2689380 10.1016/s0074-7742(08)60279-2

[B98] Eide D The SLC39 family of metal ion transporters Pflugers Arch 2004 447 796 800 10.1007/s00424-003-1074-3 12748861

[B99] Gaither LA Eide DJ The human ZIP1 transporter mediates zinc uptake in human K562 erythroleukemia cells J Biol Chem 2001 276 22258 22264 10.1074/jbc.M101772200 11301334

[B100] Palmiter RD Huang L Efflux and compartmentalization of zinc by members of the SLC30 family of solute carriers Pflugers Arch 2004 447 744 751 10.1007/s00424-003-1070-7 12748859

[B101] Liuzzi JP Cousins RJ Mammalian zinc transporters Annu Rev Nutr 2004 24 151 172 10.1146/annurev.nutr.24.012003.132402 15189117

[B102] Kambe T Yamaguchi-Iwai Y Sasaki R Nagao M Overview of mammalian zinc transporters Cell Mol Life Sci 2004 1 49 68 10.1007/s00018-003-3148-y PMC11138893 14704853

[B103] Albrecht AL Somji S Sens MA Sens DA Garrett SH Zinc transporter mRNA expression in the RWPE-1 human prostate epithelial cell line Biometals 2008 4 405 416 10.1007/s10534-007-9129-0 18097638

[B104] Lichten LA Cousins RJ Mammalian zinc transporters: nutritional and physiological regulation Annu Rev Nutr 2009 29 152 176 10.1146/annurev-nutr-033009-083312 19400752

[B105] Taylor KM A distinct role in breast cancer for two LIV-1 family zinc transporters Biochem Soc Trans 2008 36 Pt 6 1247 1251 10.1042/BST0361247 19021534

[B106] Li M Zhang Y Liu Z Bharadwaj U Wang H Wang X Zhang S Liuzzi JP Chang SM Cousins RJ Fisher WE Brunicardi FC Logsdon CD Chen C Yao Q Aberrant expression of zinc transporter ZIP4 (SLC39A4) significantly contributes to human pancreatic cancer pathogenesis and progression Proc Natl Acad Sci USA 2007 47 18636 18641 10.1073/pnas.0709307104 PMC2141829 18003899

[B107] Eide D Broderius M Fett J Guerinot ML A novel iron-regulated metal transporter from plants identified by functional expression in yeast Proc Natl Acad Sci USA 1996 1996 5624 5628 10.1073/pnas.93.11.5624 PMC39298 8643627

[B108] Zhao H Eide D The yeast ZRT1 gene encodes the zinc transporter protein of a high-affinity uptake system induced by zinc limitation Proc Natl Acad Sci USA 1996 93 2454 2458 10.1073/pnas.93.6.2454 8637895 PMC39818

[B109] Truong-Tran AQ Carter J Ruffin RE Zalewski PD The role of zinc in caspase activation and apoptotic cell death Biometals 2001 14 315 330 10.1023/A:1012993017026 11831462

[B110] Sun XM MacFarlane M Zhuang J Wolf BB Green DR Cohen GM Distinct caspase cascades are initiated in receptor-mediated and chemical-induced apoptosis J Biol Chem 1999 274 5053 5060 10.1074/jbc.274.8.5053 9988752

[B111] Strasser A O'Connor L Dixit VM Apoptosis signaling Annu Rev Biochem 2000 69 217 245 10.1146/annurev.biochem.69.1.217 10966458

[B112] Song Z Steller H Death by design: mechanism and control of apoptosis Trends Cell Biol 1999 9 49 52 10.1016/S0962-8924(99)01670-0 10611682

[B113] Thompson CB Apoptosis in the pathogenesis and treatment of disease Science 1995 267 1456 1462 10.1126/science.7878464 7878464

[B114] Wyllie AH Apoptosis: an overview Br Med Bull 1997 53 451 465 9374030 10.1093/oxfordjournals.bmb.a011623

[B115] Sensi SL Rapposelli IG Frazzini V Mascetra N Altered oxidant-mediated intraneuronal zinc mobilization in a triple transgenic mouse model of Alzheimer's disease Exp Gerontol 2008 43 5 488 92 10.1016/j.exger.2007.10.018 18068923

[B116] Maret W Molecular aspects of human cellular zinc homeostasis: redox control of zinc potentials and zinc signals Biometals 2009 22 1 149 57 10.1007/s10534-008-9186-z 19130267

[B117] Shumilina E Xuan NT Schmid E Bhavsar SK Szteyn K Gu S Götz F Lang F Zinc induced apoptotic death of mouse dendritic cells Apoptosis 2010 15 10 1177 86 10.1007/s10495-010-0520-x 20567904

[B118] Kiedaisch V Akel A Niemoeller OM Wieder T Lang F Zinc-induced suicidal erythrocyte death Am J Clin Nutr 2008 87 5 1530 4 18469280 10.1093/ajcn/87.5.1530

[B119] Sensi SL Yin HZ Carriedo SG Rao SS Weiss JH Preferential Zn2+ influx through Ca2+ -permeable AMPA/kainate channels triggers prolonged mitochondrial superoxide production Proc Natl Acad Sci USA 1999 96 2414 2419 10.1073/pnas.96.5.2414 10051656 PMC26798

[B120] Hamatake M Iguchi K Hirano K Ishida R Zinc Induces Mixed Types of Cell Death, Necrosis, and Apoptosis, in Molt-4 Cells J Biochem 2000 128 933 939 11098135 10.1093/oxfordjournals.jbchem.a022844

[B121] Untergasser G Rumpold H Plas E Witkowski M P?ster G Berger P High Levels of Zinc Ions Induce Loss of Mitochondrial Potential and Degradation of Anti-apoptotic Bcl-2 Protein in in vitro Cultivated Human Prostate Epithelial Cells Biochem Biophys Res Commun 2000 279 607 614 10.1006/bbrc.2000.3975 11118333

[B122] Bozym RA Chimienti F Giblin LJ Gross GW Korichneva I Li Y Libert S Maret W Parviz M Frederickson CJ Thompson RB Free zinc ions outside a narrow concentration range are toxic to a variety of cells in vitro Exp Biol Med 2010 235 6 741 50 10.1258/ebm.2010.009258 PMC2896872 20511678

[B123] Truong-Tran AQ Ho LH Chai F Zalewski PD Cellular zinc fluxes and the regulation of apoptosis/gene-directed cell death J Nutrition 2000 130 Suppl 1459 1466 10.1093/jn/130.5.1459S 10801960

[B124] Adamo AM Zago MP Mackenzie GG Aimo L Keen CL Keenan A Oteiza PI The role of zinc in the modulation of neuronal proliferation and apoptosis Neurotox Res 2010 17 1 1 14 10.1007/s12640-009-9067-4 19784710 PMC2797425

[B125] Chai F Truong-Tran AQ Evdokiou A Young GP Zalewski PD Intracellular Zinc Depletion Induces Caspase Activation and p21Waf1/Cip1 Cleavage in Human Epithelial Cell Lines J Infect Diseases 2000 182 S85 S92 10.1086/315914 11041715

[B126] Duvall E Wyllie AH Death and the cell Immunol Today 1986 7 115 119 10.1016/0167-5699(86)90152-0 25289803

[B127] Zalewski PD Forbes IJ Lavin M, Watters D Intracellular zinc and the regulation of apoptosis Programmed Cell Death: The Cellular and Molecular Biology of Apoptosis 1993 Melbourne: Harwood Academic Publishers

[B128] Rogers JM Taubeneck MW Daston GP Sulik KK Zucker RM Elstein KH Jankowski MA Keen CL Zinc deficiency causes apoptosis but not cell cycle alterations in organogenesis-stage rat embryos: effect of varying duration of deficiency Teratology 1995 52 149 159 10.1002/tera.1420520307 8638255

[B129] Franklin RB Costello LC The important role of the apoptotic effects of zinc in the development of cancers Journal of Cellular Biochemistry 2009 106 750 757 10.1002/jcb.22049 19160419 PMC2727867

[B130] Peterson QP Goode DR West CW Ramsey KN Lee JJY Hergenrother PJ PAC-1 activates procaspase-3 in vitro through relief of zinc-mediated inhibition J Mol Biol 2009 388 144 158 10.1016/j.jmb.2009.03.003 19281821 PMC2714579

[B131] Oteiza PI Olin KL Fraga CG Keen CL Zinc deficiency causes oxidative damage to proteins, lipids and DNA in rat testes J Nutr 1995 125 823 829 7722683 10.1093/jn/125.4.823

[B132] Taylor CG Towner RA Janzen EG Bray TM MRI detection of hyperoxiainduced lung edema in Zn deficient rats Free Radic Biol Med 1990 9 229 233 10.1016/0891-5849(90)90033-F 2272531

[B133] Kraus A Roth HP Kirchgessner M Supplementation with vitamin C, vitamin E or beta-carotene influences osmotic fragility and oxidative damage of erythrocytes of zinc-deficient rats J Nutr 1997 127 1290 1296 9202082 10.1093/jn/127.7.1290

[B134] Zalewski PD Forbes IJ Betts WH Correlation of apoptosis with change in intracellular labile Zn, using Zinquin, a new specific fluorescent probe for zinc Biochem J 1993 296 403 408 8257431 10.1042/bj2960403 PMC1137710

[B135] Meerarani P Ramadass P Toborek M Bauer HC Bauer H Hennig B Zinc protects against apoptosis of endothelial cells induced by linoleic acid and tumor necrosis factor alpha Am J Clin Nutr 2000 71 81 87 10617950 10.1093/ajcn/71.1.81

[B136] Vallee BL Falchuk KH The biochemical basis of zinc physiology Physiol Rev 1993 79 118 8419966 10.1152/physrev.1993.73.1.79

[B137] Yang Y Kawataki T Fukui K Koike T Cellular Zn2+ chelators cause "dying-back" neurite degeneration associated with energy impairment J Neurosci Res 2007 85 13 2844 55 10.1002/jnr.21411 17628505

[B138] Baird SK Kurz T Brunk UT Metallothionein protects against oxidative stress-induced lysosomal destabilization Biochem J 2006 394 Pt 1 275 83 16236025 10.1042/BJ20051143 PMC1386026

[B139] Williams RJP The biochemistry of zinc Polyhedron 1987 6 61 69 10.1016/S0277-5387(00)81239-5

[B140] Roychowdhury M Sarkar N Manna T Bhattacharyya S Sarkar T Basusarkar P Roy S Bhattacharyya B Sulfhydryls of tubulin: A probe to detect conformational changes of tubulin Eur J Biochem 2000 267 3469 3476 10.1046/j.1432-1327.2000.01369.x 10848962

[B141] Hesketh JE Zinc-stimulated microtubule assembly and evidence for zinc binding to tubulin Int J Biochem 1982 14 983 990 10.1016/0020-711X(82)90059-3 7141075

[B142] Banan A Fields JZ Decker H Zhang Y Keshavarzian A Nitric oxide and its metabolites mediate ethanol-induced microtubule disruption and intestinal barrier dysfunction J Pharmacol Exp Ther 2000 294 997 1008 10945852

[B143] Martin SJ Cotter TG Specific loss of microtubules in HL-60 cells leads to programmed cell death (apoptosis) Biochem Soc Trans 1990 18 299 301 2379727 10.1042/bst0180299a

[B144] Bozym RA Thompson RB Stoddard AK Fierke CA Measuring picomolar intracellular exchangeable zinc in PC-12 cells using a ratiometric fluorescence biosensor ACS Chem Biol 2006 1 2 103 11 10.1021/cb500043a 17163650

[B145] Floersheim GL Christ A Koenig R Racine C Gudat F Radiation-induced lymphoid tumors and radiation lethality are inhibited by combined treatment with small doses of zinc aspartate and WR 2721 Int J Cancer 1992 52 604 608 10.1002/ijc.2910520419 1328072

[B146] Matsushita K Kitagawa K Matsuyama T Ohtsuki T Taguchi A Mandai K Mabuchi T Yagita Y Yanagihara T Matsumoto M Effect of systemic zinc administration on delayed neuronal death in the gerbil hippocampus Brain Res 1996 743 362 365 10.1016/S0006-8993(96)01112-2 9017270

[B147] Kuo IC Seitz B LaBree L McDonnell PJ Can zinc prevent apoptosis of anterior keratocytes after superfcial keratectomy Cornea 1997 16 550 555 10.1097/00003226-199709000-00011 9294688

[B148] Sankaramanivel S Rajaram A Rajaram R Zinc protects human peripheral blood lymphocytes from Cr(III)(phenanthroline)3-induced apoptosis Toxicol Appl Pharmacol 2010 243 3 405 19 10.1016/j.taap.2009.12.018 20043934

[B149] Sunderman FW The influence of zinc on apoptosis Ann Clin Lab Sci 1995 25 134 142 7785963

[B150] Raymond AD Gekonge B Giri MS Hancock A Papasavvas E Chehimi J Kossevkov AV Nicols C Yousef M Mounzer K Shull J Kostman J Showe L Montaner LJ Increased metallothionein gene expression, zinc, and zinc-dependent resistance to apoptosis in circulating monocytes during HIV viremia J Leukoc Biol 2010 88 3 589 96 10.1189/jlb.0110051 20551211 PMC2924602

[B151] Zalewski PD Forbes IJ Giannakis C Physiological role for zinc in prevention of apoptosis (gene-directed death) Biochem Inter 1991 24 1093 1101 1781788

[B152] Kolenko V Uzzo RG Bukowski R Bander NH Novick AC His ED Finke JH Dead or dying: necrosis versus apoptosis in caspase-deficient human renal cell carcinoma Cancer Res 1999 59 2838 2842 10383143

[B153] Yorimitsu T Klionsky DJ Autophagy: molecular machinery for self-eating Cell Death Differ 2005 12 Suppl2 1542 1552 10.1038/sj.cdd.4401765 16247502 PMC1828868

[B154] Majeski AE Dice JF Mechanisms of chaperone-mediated autophagy Int J Biochem Cell Biol 2004 36 2435 2444 10.1016/j.biocel.2004.02.013 15325583

[B155] Massey A Kiffin R Cuervo AM Pathophysiology of chaperone-mediated autophagy Int J Biochem Cell Biol 2004 36 2420 2434 10.1016/j.biocel.2004.04.010 15325582

[B156] Reggiori F Klionsky DJ Autophagy in the eukaryotic cell Eukaryot Cell 2002 1 11 21 10.1128/EC.01.1.11-21.2002 12455967 PMC118053

[B157] Wang C-W Klionsky DJ The molecular mechanism of autophagy Mol Med 2003 9 65 76 12865942 PMC1430730

[B158] Kuma A Hatano M Matsui M Yamamoto A Nakaya H Yoshimori T Ohsumi Y Tokuhisa T Mizushima N The role of autophagy during the early neonatal starvation period Nature 2004 432 1032 1036 10.1038/nature03029 15525940

[B159] Mizushima N Yamamoto A Matsui M Yoshimori T Ohsumi Y vivo analysis of autophagy in response to nutrient starvation using transgenic mice expressing a fluorescent autophagosome marker Mol Biol Cell 2004 15 1101 1111 10.1091/mbc.E03-09-0704 14699058 PMC363084

[B160] Levine B Klionsky DJ Development by self-digestion: molecular mechanisms and biological functions of autophagy Dev Cell 2004 6 463 477 10.1016/S1534-5807(04)00099-1 15068787

[B161] Cuervo AM Autophagy: in sickness and in health Trends Cell Biol 2004 14 70 77 10.1016/j.tcb.2003.12.002 15102438

[B162] Shintani T Klionsky DJ Autophagy in health and disease: a double-edged sword Science 2004 306 990 995 10.1126/science.1099993 15528435 PMC1705980

[B163] Kirkegaard K Taylor MP Jackson WT Cellular autophagy: surrender, avoidance and subversion by microorganisms Nat Rev Microbiol 2004 2 301 314 10.1038/nrmicro865 15031729 PMC7097095

[B164] Levine B Eating oneself and uninvited guests: autophagy-related pathways in cellular defense Cell 2005 120 159 162 15680321 10.1016/j.cell.2005.01.005

[B165] Bursch W Multiple cell death programs: Charon's lifts to Hades FEMS Yeast Res 2004 5 101 110 10.1016/j.femsyr.2004.07.006 15489192

[B166] Lee SJ Cho KS Koh JY Oxidative injury triggers autophagy in astrocytes: the role of endogenous zinc Glia 2009 57 351 361 10.1002/glia.20763 19229997

[B167] Chung H Yoon YH Hwang JJ Cho KS Koh JY Kim JG Ethambutol-induced toxicity is mediated by zinc and lysosomal membrane permeabilization in cultured retinal cells Toxicol Appl Pharmacol 2009 235 163 170 10.1016/j.taap.2008.11.006 19063910

[B168] Iwanyshyn WM Han GS Carman GM Regulation of phospholipid synthesis in Saccharomyces cerevisiae by zinc J Biol Chem 2004 279 21 21976 83 10.1074/jbc.M402047200 15028711

[B169] Hwang JJ Kim HN Kim J Cho DH Kim MJ Kim YS Kim Y Park SJ Koh JY Zinc(II) ion mediates tamoxifen-induced autophagy and cell death in MCF-7 breast cancer cell line Biometals 2010 23 6 997 1013 10.1007/s10534-010-9346-9 20524045

[B170] Hwang JJ Lee SJ Kim TY Cho JH Koh JY Zinc and 4-hydroxy-2-nonenal mediate lysosomal membrane permeabilization induced by H2O2 in cultured hippocampal neurons J Neurosci 2008 28 12 3114 22 10.1523/JNEUROSCI.0199-08.2008 18354014 PMC6670692

[B171] Lee SJ Park MH Kim HJ Koh JY Metallothionein-3 regulates lysosomal function in cultured astrocytes under both normal and oxidative conditions Glia 2010 58 10 1186 96 20544854 10.1002/glia.20998

[B172] Lee HK Mattei LM Steinberg BE Alberts P Lee YH Chervonsky A Mizushima N Grinstein S Iwasaki A In vivo requirement for Atg5 in antigen presentation by dendritic cells Immunity 2010 32 2 227 39 10.1016/j.immuni.2009.12.006 20171125 PMC2996467

[B173] Sparvero LJ Asafu-Adjei D Kang R Tang D Amin N Im J Rutledge R Lin B Amoscato AA Zeh HJ Lotze MT RAGE (Receptor for Advanced Glycation Endproducts), RAGE ligands, and their role in cancer and inflammation J Transl Med 2009 17 10.1186/1479-5876-7-17 19292913 PMC2666642

[B174] Lotze MT Tracey KJ High-mobility group box 1 protein (HMGB1): nuclear weapon in the immune arsenal Nat Rev Immunol 2005 5 331 342 10.1038/nri1594 15803152

[B175] Tang DL Kang R Zeh HJ Lotze MT HMGB1 and Cancer Biochim Biophys Acta 2010 1799 1-2 131 40 20123075 10.1016/j.bbagrm.2009.11.014 PMC2818552

[B176] Prasad AS Effects of zinc deficiency on Th1 and Th2 cytokine shifts J Infect Dis 2000 2182 Suppl 1 62 68 10.1086/315916 10944485

[B177] Tanaka S Akaishi E Hosaka K Okamura S Kubohara Y Zinc ions suppress mitogen-activated interleukin-2 production in Jurkat cells Biochem Biophys Res Commun 2005 335 1 162 7 10.1016/j.bbrc.2005.07.059 16055081

[B178] Wu W Silbajoris RA Cao D Bromberg PA Zhang Q Peden DB Samet JM Regulation of cyclooxygenase-2 expression by cAMP response element and mRNA stability in a human airway epithelial cell line exposed to zinc Toxicol Appl Pharmacol 2008 231 2 260 6 10.1016/j.taap.2008.04.012 18513776

[B179] Shen H Oesterling E Stromberg A Toborek M MacDonald R Hennig B Zinc deficiency induces vascular pro-inflammatory parameters associated with NF-kappaB and PPAR signaling J Am Coll Nutr 2008 27 5 577 87 18845708 10.1080/07315724.2008.10719741

[B180] Yamaki K Yoshino S Comparison of inhibitory activities of zinc oxide ultrafine and fine particulates on IgE-induced mast cell activation Biometals in press Available online, 2009 Jul 17 19609684 10.1007/s10534-009-9254-z

[B181] Putics A Vödrös D Malavolta M Mocchegiani E Csermely P Soti C Zinc supplementation boosts the stress response in the elderly: Hsp70 status is linked to zinc availability in peripheral lymphocytes Exp Gerontol 2008 43 5 452 61 10.1016/j.exger.2008.01.002 18304769

[B182] Safieh-Garabedian B Poole S Allchorne A Kanaan S Saade N Woolf CJ Zinc reduces the hyperalgesia and upregulation of NGF and IL-1 beta produced by peripheral inflammation in the rat Neuropharmacology 1996 35 5 599 603 10.1016/0028-3908(96)84630-2 8887968

[B183] Aydemir TB Liuzzi JP McClellan S Cousins RJ Zinc transporter ZIP8 (SLC39A8) and zinc influence IFN-gamma expression in activated human T cells J Leukoc Biol 2009 86 2 337 48 10.1189/jlb.1208759 19401385 PMC2726764

[B184] Bao B Prasad A Beck FW Suneja A Sarkar F Toxic effect of zinc on NF-kappaB, IL-2, IL-2 receptor alpha, and TNF-alpha in HUT-78 (Th(0)) cells Toxicol Lett 2006 166 3 222 8 10.1016/j.toxlet.2006.07.306 16930873

[B185] Mariani E Neri S Cattini L Mocchegiani E Malavolta M Dedoussis GV Kanoni S Rink L Jajte J Facchini A Effect of zinc supplementation on plasma IL-6 and MCP-1 production and NK cell function in healthy elderly: interactive influence of +647 MT1a and -174 IL-6 polymorphic alleles Exp Gerontol 2008 43 5 462 71 10.1016/j.exger.2007.12.003 18215484

[B186] Giacconi R Cipriano C Muti E Costarelli L Maurizio C Saba V Gasparini N Malavolta M Mocchegiani E Novel -209A/G MT2A polymorphism in old patients with type 2 diabetes and atherosclerosis: relationship with inflammation (IL-6) and zinc Biogerontology 2005 6 6 407 13 10.1007/s10522-005-4907-y 16518702

[B187] Rajagopalan S Winter CC Wagtmann N Long EO The Ig-related killer cell inhibitory receptor binds zinc and requires zinc for recognition of HLA-C on target cells J Immunol 1995 155 9 4143 6 7594568

[B188] Valés-Gómez M Erskine RA Deacon MP Strominger JL Reyburn HT The role of zinc in the binding of killer cell Ig-like receptors to class I MHC proteins Proc Natl Acad Sci USA 2001 98 4 1734 9 10.1073/pnas.041618298 11172020 PMC29326

[B189] Li Y Li H Dimasi N McCormick JK Martin R Schuck P Schlievert PM Mariuzza RA Crystal structure of a superantigen bound to the high-affinity, zinc-dependent site on MHC class II Immunity 2001 14 1 93 104 10.1016/S1074-7613(01)00092-9 11163233

[B190] Roussel A Anderson BF Baker HM Fraser JD Baker EN Crystal structure of the streptococcal superantigen SPE-C: dimerization and zinc binding suggest a novel mode of interaction with MHC class II molecules Nat Struct Biol 1997 4 8 635 43 10.1038/nsb0897-635 9253413

[B191] Prasad AS Bao B Beck FW Sarkar FH Zinc activates NF-kappaB in HUT-78 cells J Lab Clin Med 2001 138 4 250 6 10.1067/mlc.2001.118108 11574819

[B192] Shifera AS Horwitz MS Mutations in the zinc finger domain of IKK gamma block the activation of NF-kappa B and the induction of IL-2 in stimulated T lymphocytes Mol Immunol 2008 45 6 1633 45 10.1016/j.molimm.2007.09.036 18207244

[B193] Kim I Kim CH Kim JH Lee J Choi JJ Chen ZA Lee MG Chung KC Hsu CY Ahn YS Pyrrolidine dithiocarbamate and zinc inhibit proteasome-dependent proteolysis Exp Cell Res 2004 298 1 229 38 10.1016/j.yexcr.2004.04.017 15242777

[B194] Jarrousse V Castex-Rizzi N Khammari A Charveron M Dréno B Zinc salts inhibit in vitro Toll-like receptor 2 surface expression by keratinocytes Eur J Dermatol 2007 17 6 492 6 17951128 10.1684/ejd.2007.0263

[B195] O'Reilly SM Moynagh PN Regulation of Toll-like receptor 4 signalling by A20 zinc finger protein Biochem Biophys Res Commun 2003 303 2 586 93 10.1016/S0006-291X(03)00389-9 12659860

[B196] Zhang W Mi J Li N Sui L Wan T Zhang J Chen T Cao X Identification and characterization of DPZF, a novel human BTB/POZ zinc finger protein sharing homology to BCL-6 Biochem Biophys Res Commun 2001 282 4 1067 73 10.1006/bbrc.2001.4689 11352661

[B197] Sharif-Askari E Vassen L Kosan C Khandanpour C Gaudreau MC Heyd F Okayama T Jin J Rojas ME Grimes HL Zeng H Möröy T Zinc finger protein Gfi1 controls the endotoxin-mediated Toll-like receptor inflammatory response by antagonizing NF-kappaB p65 Mol Cell Biol 2010 30 16 3929 42 10.1128/MCB.00087-10 20547752 PMC2916436

[B198] Jin J Zeng H Schmid KW Toetsch M Uhlig S Möröy T The zinc finger protein Gfi1 acts upstream of TNF to attenuate endotoxin-mediated inflammatory responses in the lung Eur J Immunol 2006 36 2 421 30 10.1002/eji.200535155 16402406

[B199] Lademann U Kallunki T Jäättelä M A20 zinc finger protein inhibits TNF-induced apoptosis and stress response early in the signaling cascades and independently of binding to TRAF2 or 14-3-3 proteins Cell Death Differ 2001 8 3 265 72 10.1038/sj.cdd.4400805 11319609

[B200] Hong JW Allen CE Wu LC Inhibition of NF-kappaB by ZAS3, a zinc-finger protein that also binds to the kappaB motif Proc Natl Acad Sci USA 2003 100 21 12301 6 10.1073/pnas.2133048100 14530385 PMC218753

[B201] Lin LC Que J Lin KL Leung HW Lu CL Chang CH Effects of zinc supplementation on clinical outcomes in patients receiving radiotherapy for head and neck cancers: a double-blinded randomized study Int J Radiat Oncol Biol Phys 2008 70 2 368 73 10.1016/j.ijrobp.2007.06.073 17980503

[B202] Yamaguchi S Subtraction cloning of growth arrest inducible genes in normal human epithelial cells Kokubyo Gakkai Zasshi 1995 62 78 93 7751801 10.5357/koubyou.62.78

[B203] Desouki MM Geradts J Milon B Franklin RB Costello LC hZip2 and hZip3 zinc transporters are down regulated in human prostate adenocarcinomatous glands Mol Cancer 2007 6 37 10.1186/1476-4598-6-37 17550612 PMC1892035

[B204] Kagara N Tanaka N Noguchi S Hirano T Zinc and its transporter ZIP10 are involved in invasive behavior of breast cancer cells Cancer Sci 2007 5 692 697 10.1111/j.1349-7006.2007.00446.x PMC11159674 17359283

[B205] Cousins RJ Liuzzi JP Lichten LA Mammalian zinc transport, trafficking, and signals J Biol Chem 2006 281 24085 24089 10.1074/jbc.R600011200 16793761

[B206] Taylor KM Vichova P Jordan N Hiscox S Hendley R Nicholson RI ZIP7 mediated intracellular zinc transport contributes to aberrant growth factor signaling in antihormone-resistant breast cancer Cells Endocrinology 2008 149 10 4912 4920 10.1210/en.2008-0351 18583420

[B207] Hogstrand C Kille P Nicholson RI Taylor KM Zinc transporters and cancer: a potential role for ZIP7 as a hub for tyrosine kinase activation Trends Mol Med 2009 15 3 101 111 10.1016/j.molmed.2009.01.004 19246244

